# Extreme flood return levels in a U.S. mid-Atlantic estuary using 40-year fluvial-coastal model simulations

**DOI:** 10.1038/s41597-025-05566-9

**Published:** 2025-08-21

**Authors:** Mithun Deb, Ning Sun, Zhaoqing Yang, Taiping Wang, David Judi, Matthew G. Cooper, Mark S. Wigmosta

**Affiliations:** 1 Marine and Coastal Research Laboratory, Energy and Environment Directorate, Pacific Northwest National Laboratory, Sequim, WA 98382 USA; 2 Energy and Environment Directorate, Pacific Northwest National Laboratory, Richland, WA 99354 USA; 3 Department of Civil and Environmental Engineering, University of Washington, Seattle, WA 98195 USA; 4 Atmospheric, Climate, and Earth Sciences Division, Pacific Northwest National Laboratory, Richland, WA 99354 USA

**Keywords:** Natural hazards, Physical oceanography

## Abstract

Using an integrated watershed-coastal modeling framework, we conducted long-term historical simulations (1980-2019) of fluvial and coastal flooding in the Delaware Bay and River, a vulnerable estuarine system in the U.S., at high spatial resolutions. By applying process-based models, we generated physically consistent and spatially detailed estimates of estuarine, riverine, and surge-driven extreme water level compared to previous studies that used field data only. We then evaluated changes in the magnitude of flood events using the 40-year simulations and detrended Floodwater Depth values with stationary extreme value analysis. Our detailed assessment of spatial-varying extreme values revealed how different flood-generation mechanisms can dominate various zones in the estuary. The datasets produced through this work will be valuable for long-term flood hazard mitigation planning in coastal communities in the Delaware Bay and River region. Additionally, this work will serve as a benchmark for other coastal flood hazard modeling communities worldwide, aiding them in systematically modeling long-term and continuous extreme flood events.

## Background & Summary

Globally, coastal cities nowadays face increasing flood risks due to climate change, relative sea level rise, and changes in hurricane intensity and frequency^1–4^. Based on observations and model ensembles covering 5,000 coastal regions and major cities worldwide, Climate Impact Lab and the UNDP’s Human Development Report Office^5^ reported that by 2050, 5% of coastal city populations will be exposed to higher flood risk and the number could double by 2100 (nearly 73 million people), significantly affecting social and economic hubs and leading to global reversals in human development. Since the 1950s, flooding has increased in cities near the U.S. coastline, with the East and Gulf Coasts experiencing the largest increase in flood frequency^6^. Although storm surges pose a significant threat to human lives^7^, they can also cause various other damages, such as road closures, blocking of stormwater drainage and facilitation of pluvial flooding, loss of infrastructure and public services, environmental and economic impacts, and more^8^. For coastal cities in the U.S., these damages are particularly alarming, as there are nearly $1 trillion worth of property and structures, along with more than 40% of the population living near the coast^9^. Some of the notable extreme hurricane events in the last decade, Hurricane Maria (2017), Hurricane Harvey (2017), and Hurricane Ian (2022), caused an estimated $111.6B, $155.0B, and $116.3B worth of total damage, respectively^10^. This trend has continued with more recent events, including Hurricane Helene (2024) and Hurricane Milton (2024), which caused estimated damage of $78.7B and $34.7B, further emphasizing the increasing impact of extreme weather events^11^. Flood managers, decision makers, and planners often rely on extreme flood return levels [based on Annual Exceedance Probability (AEP)] to prepare for and respond to such disasters–ultimately to reduce the human, economic, and environmental impacts during extreme events worldwide^12–14^. Knowing the flood magnitude for different recurrence intervals, such as the 1-percent AEP or 100-year flood, helps manage risks to life and property, design infrastructure, plan insurance and finances, manage floodplains, ensure regulatory compliance, and more. In the United States, government agencies like the Federal Emergency Management Agency (FEMA^15^), U.S. Geological Survey (USGS^12^), and U.S. Army Corps of Engineers (USACE^16^), in addition to individual state agencies, use flood return level datasets to support their services related to flood risk management and decision-making processes.

Although the extreme flood return level is an essential variable for various agencies managing large coastal/estuarine regions, estimating its value using statistical methods is not trivial due to the recorded data’s lack of temporal and spatial coverage. For many coastal areas of the U.S. and worldwide, it is primarily projected using long-term water level observations from tide gauge locations, which are often sparsely distributed over a large region^17–20^. To address this issue, recent regional and global studies focused on coastlines have employed statistical methods like Regional Frequency Analysis^21,22^ and Bayesian Hierarchical Models^23^. These techniques provide spatial estimates of extreme sea levels in locations without tide gauges, utilizing the limited available data from real gauges. Simultaneously, various hydrodynamic modeling frameworks have been utilized over the last decade to accommodate many critical issues with using field data, such as gaps in continuous observation, insufficient data period, and unavailability in the place of interest^24,25^. Even though numerical models can significantly mitigate these limitations, the reliability of statistical estimates derived from model simulations depends on their accuracy, as well as spatial and temporal coverage. Therefore, to obtain more accurate estimates of extreme flood return levels in coastal and estuarine regions – which are affected by interacting coastal and river processes – an integrated high-resolution numerical modeling framework that can capture key physical and non-linear interactions between atmospheric, hydrologic, and hydrodynamic processes is essential.

**Fig. 1:**
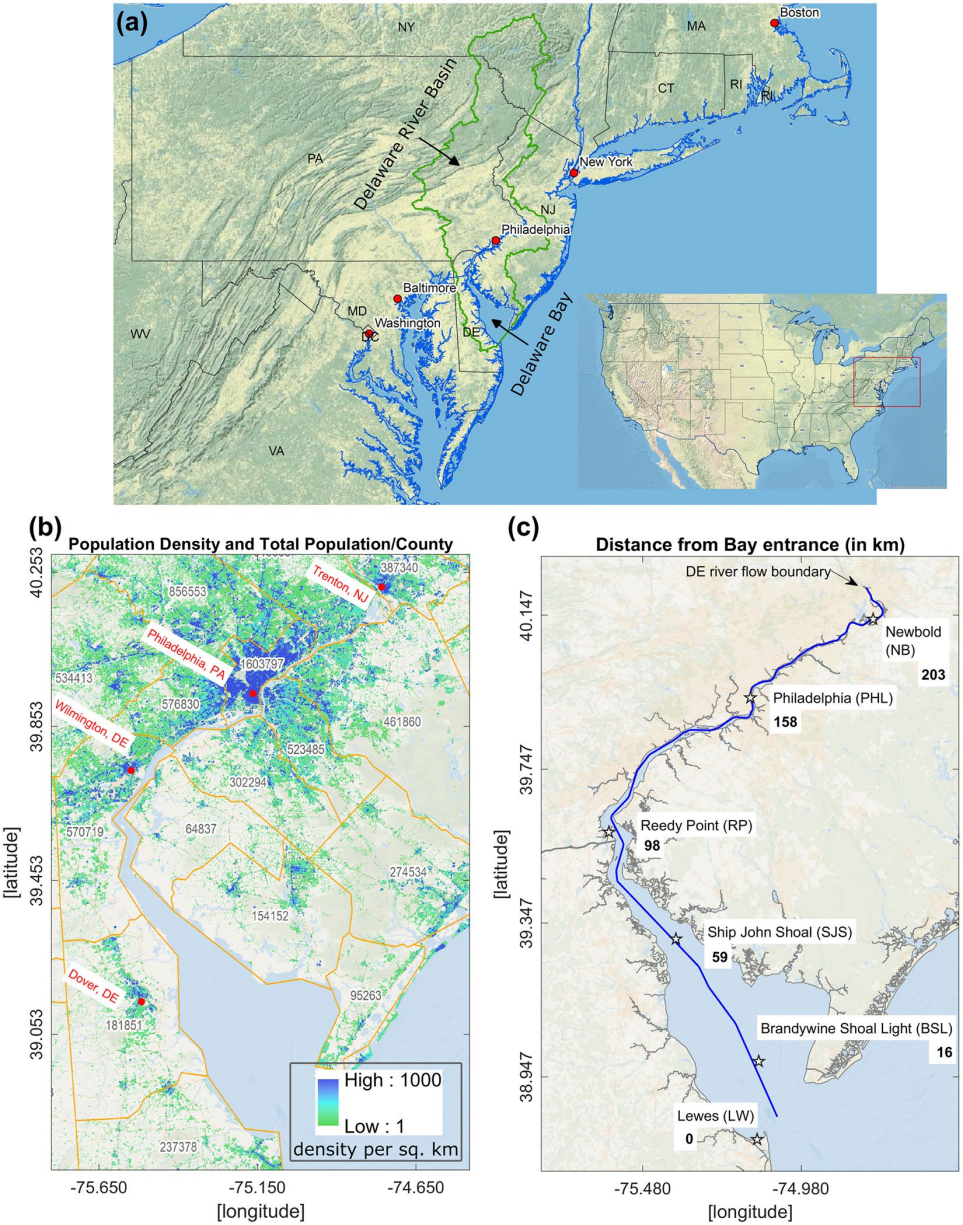
(**a**) Study area: Delaware Bay and River (DBR) in the U.S. Mid-Atlantic. The blue line represents the shoreline, and the green line represents the Delaware River Basin; (**b**) A zoomed-in view of the bay and river. The LandScan USA (2021) population density data (1-km resolution) and the distribution of the total county population are added to the map; (**c**) Map showing the channel thalweg transect and distance from the entrance of the bay, along with the National Oceanic and Atmospheric Administration (NOAA) tide gauge locations used for model validation.

In this work, we implement a combined deterministic and statistical modeling approach for estimating the spatial variation of extreme flood return levels in Delaware Bay and River (DBR), a large U.S. mid-Atlantic estuary (Fig. 1). The Delaware River Basin houses nearly 14.2 million people and annually contributes about $22 billion in economic activity to the region. The tidally-dominated estuarine portion of the basin, which also consists of a major U.S. city, Philadelphia, is highly vulnerable to flood hazards due to a combination of factors, including its converging (funnel-like) shape, the impacts of climate change, and the destructive hurricanes that make landfall or translates through the area^26^. This is also a critical region where interacting fluvial and coastal flood events can generate compound flooding^27^. These compound events are particularly challenging to predict, making integrated modeling framework applied in this work necessary for estimating extreme flood magnitudes.

To address these challenges, we integrate two high-resolution, physics-based models to simulate both riverine and coastal processes across the DBR. The Distributed Hydrology Soil Vegetation Model (DHSVM^28^) is used to simulate watershed hydrology and fluvial flooding, while the Finite Volume Community Ocean Model (FVCOM^29^) is used to model coastal hydrodynamics. Together, these models performed simulations from 1980 to 2019, enabling the assessment of various flood events over four decades. The continuous meteorological forcing for the historical climate is obtained from a dynamically downscaled ECMWF Reanalysis v5 (ERA5) dataset, called ‘WRF-TGW’^30^. After rigorous model calibration and validation, we estimated the changes to the magnitude of the Floodwater Depth [observed water level - the maximum predicted tide (each year)] over time using the 40-year continuous water level dataset and a Python-based stationary extreme value analysis tool called *PyExtreme* (link is provided in the Code availability section). Subsequently, two classical statistical methods: 1) Generalized Extreme Value distribution with Block Maxima fit^31^ (BM/GEV) and 2) Points-Over-Threshold fit to Generalized Pareto distribution^31^ (POT/GPD) are used to estimate the Floodwater Depth return levels. We implemented a step-by-step approach to verify the performance of these statistical methods in predicting Floodwater Depth magnitude for different recurrence intervals using observation and model data. Results show that the performance of these statistical methods is sensitive to the location of the estuary. The BM/GEV approach provides a better fit and smaller confidence interval near the open coast than POT/GPD, where the flooding is storm surge-dominated and less frequent. Conversely, the POT/GPD approach performs better in the river portion, with frequent fluvial flooding and much more significant data points. We generated return value estimates for different recurrence intervals along with the 95% confidence intervals for the entire study area using both methods. Providing results from both approaches allows users to evaluate how the choice of method impacts the estimates’ magnitude and uncertainty. This flexibility helps users select the appropriate method for their specific needs, such as infrastructure design, flood risk assessment, or policy development, while considering the statistical properties of the data, such as record length and frequency of flood events. It will also be a valuable dataset for the flood management communities in DBR because of its extensive spatial coverage and high resolution compared to traditional estimates based on single-point tide gauge datasets. Additionally, a dataset of simulated water levels and extreme flood return levels could help understand the distribution, trend, and variability of different flood processes over the long term. Ultimately, such a flood dataset can capture fundamental human-natural system dynamics and help simulate time-evolving risks within the region.

## Methods

### Methods overview

**Fig. 2:**
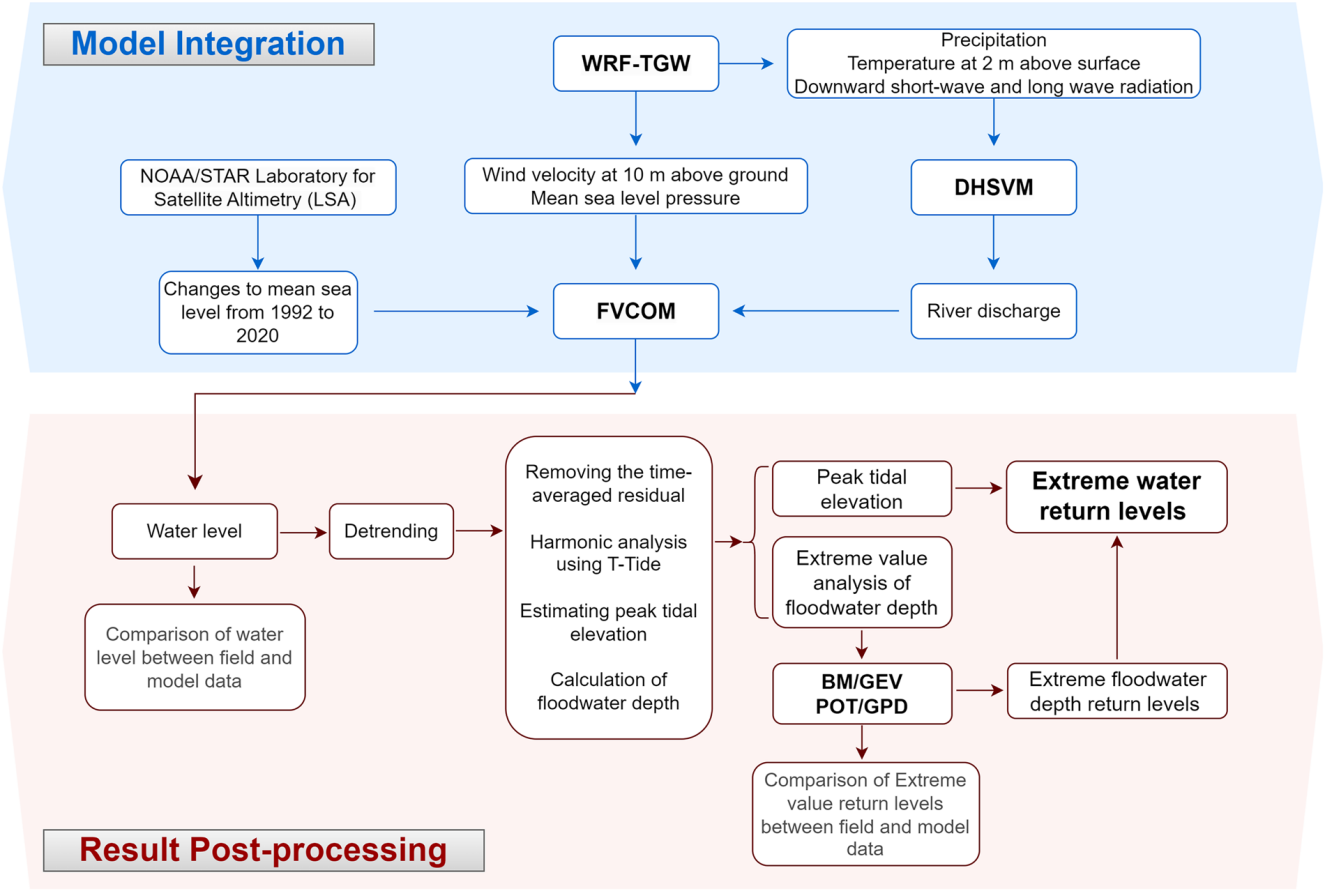
Sketch showing the process of data collection, model input file preparation, hydrodynamic model result post-processing, and finally using the dataset for statistical analysis and extreme water return level estimates.

At the onset, we collected the dynamically downscaled atmospheric re-analysis data that covers 40 years (1980 to 2019) from the WRF-TGW dataset to provide continuous meteorological forcing for the higher-resolution hydrology and hydrodynamic models. We demonstrate this process in Fig. 2, where the hydrological processes are simulated first using DHSVM to provide river forcing for the hydrodynamic model. Then, FVCOM uses the river forcing, the meteorological forcing from WRF-TGW, and water surface elevation at the open ocean boundary to provide continuous 40-year water level data for the entire model domain covering DBR. The links for obtaining both these models are provided in the Code availability section. The outputs from both DHSVM and FVCOM models underwent extensive validation against observed datasets to evaluate model skills before their application in further analysis. Following model validation, we identified continuous Floodwater Depth magnitudes using data detrending, tide averaging, and tidal harmonic analysis on the full simulation period. The second phase of the study focuses on characterizing extremes through statistical modeling. Specifically, we implement two well-established extreme value approaches, (a) BM/GEV and (b) POT/GPD, to estimate return levels of Floodwater Depth across the entire region. These methods are detailed in the subsections that follow.

### Atmospheric reanalysis data from 1980 to 2019 (WRF-TGW)

**Fig. 3:**
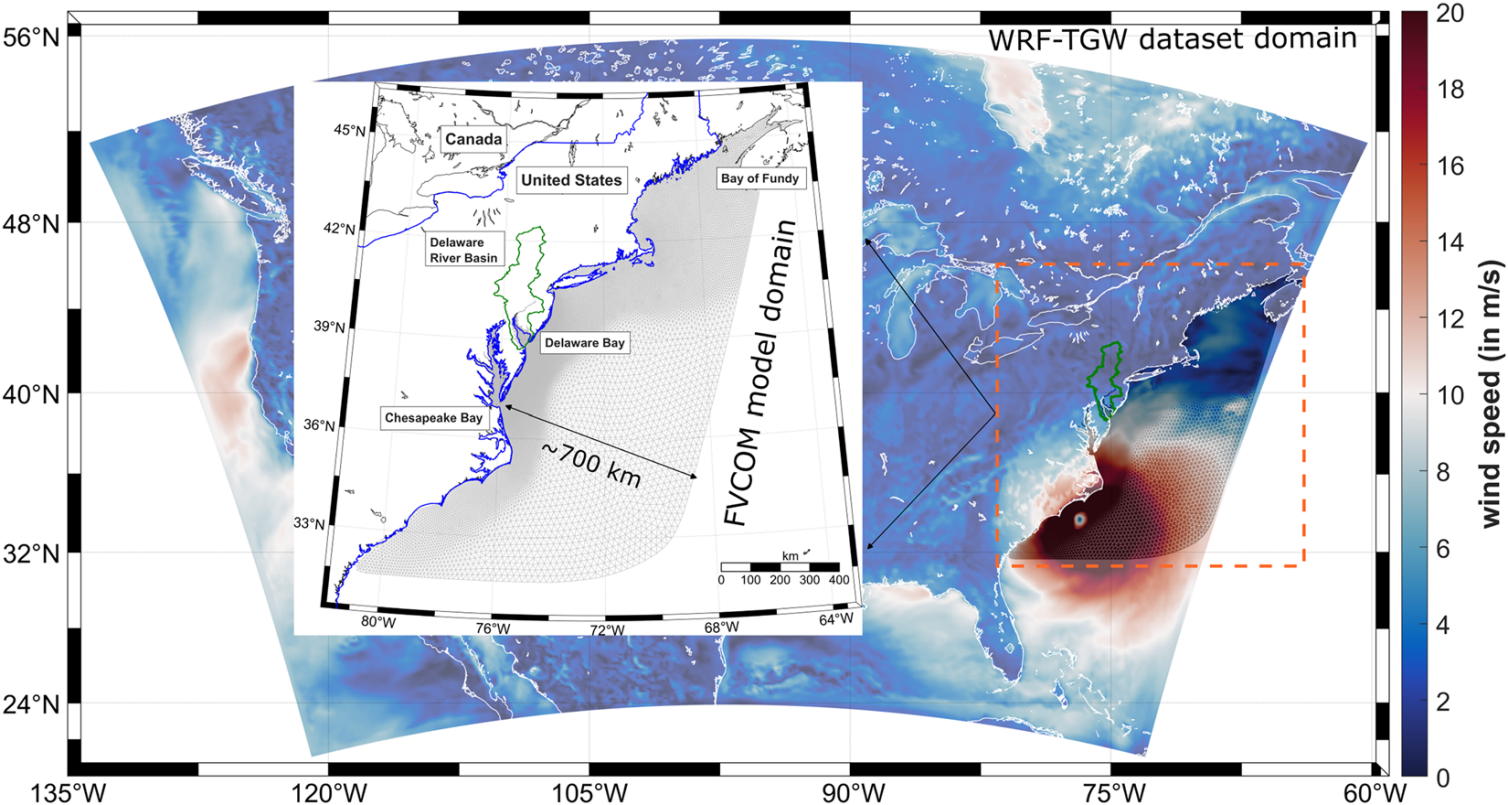
Atmospheric dataset ‘WRF-TGW’ domain covering the Continental United States. The colormap represent the 10-m elevation wind speed during Hurricane Irene (2011) landfall in the U.S. Mid-Atlantic; The top subplot shows the hydrology (DHSVM) and hydrodynamic (FVCOM) model domains.

To explore the potential impact of climate change on weather patterns, the Department of Energy’s IM3 and HyperFACETS projects have developed an atmospheric dataset called WRF-TGW^30^. This dataset covers the Continental United States (CONUS), northern Mexico, and southern Canada. We use the 40-year sequence of past weather from 1980 to 2019, which is the European Centre for Medium-Range Weather Forecasts Reanalysis v5 (ECMWF ERA5) data (30 km resolution), dynamically downscaled with the Weather Research and Forecasting (WRF) model to achieve a horizontal resolution of approximately 12 km. This higher resolution is crucial for resolving flood processes in complex estuarine regions as it captures regional atmospheric processes at finer scales such as extreme event precipitation and wind fields^26^. In addition to the finer resolution, the WRF-TGW dataset domain extends approximately 700 km offshore from the US Mid-Atlantic coastline (shown in Fig. 3), providing a reasonable area coverage for representing the hurricane field that affected the DBR. We use this historical reanalysis data to simulate the sequence of flood events from 1980 to 2019. To verify the skill of this dataset, we collected wind speed data from multiple near-shore National Data Buoy Center (NDBC) buoys during Hurricane Irene (2011) and Hurricane Sandy (2012) and observed a good agreement between the downscaled data and the field observation. From this dataset, we utilized temperature, precipitation, wind speed, relative humidity, and downward short-wave and long-wave radiation at ground level to force DHSVM, along with wind velocity at 10 meters and sea surface pressure as surface forcing for FVCOM.

### Numerical models for riverine and coastal flood modeling

#### DHSVM

DHSVM is a hydrological model that simulates key overland and subsurface processes by explicitly representing climate, soil, and vegetation at the grid cell level. It is a physics-based, spatially distributed hydrological model where it solves the full energy and water balance equations. The model comprises several components, including a two-layer canopy model for evapotranspiration, a two-layer snowpack model for snow accumulation and melt, a multi-layer unsaturated soil model that moves infiltrated water vertically based on Darcy’s law, and a saturated subsurface flow routing model that uses a transient quasi three-dimensional model. The excess surface runoff and intercepted subsurface flow routes through the stream network to the watershed outlet, and the channel flow conveyance uses segments of linear reservoirs. More information about model physics and formulations is detailed in the existing literature^28,32,33^. For this study, DHSVM was configured for the Delaware River Basin at a 90-m spatial resolution. In addition to meteorological inputs from WRF-TGW, key geospatial inputs for DHSVM include digital elevation model (DEM), vegetation data from USGS National Land Cover Database, and soil data from U.S. Department of Agriculture (USDA) National Resources Conservation Service State Soil Geographic (STATSGO), all resampled to a 90-m grid resolution. A pre-processing tool was used to generate the Delaware River network based on DEM, resulting in 6225 river segments across the Delaware River network. For each river segment, DHSVM generated outputs of river discharge at a 3-hourly timestep. The river forcing used by FVCOM includes 13 reaches at the river-ocean interface (i.e., DHSVM-FVCOM domain), with the major ones being the Schuylkill and Delaware mainstem near Trenton, NJ.

#### FVCOM

Using atmospheric forcing from the WRF-TGW dataset and river forcing from DHSVM, we simulate the water surface elevation (WSE) using unstructured grid FVCOM^29^. It is extensively used to model storm surges and flooding in many estuarine and coastal regions, especially with complex bathymetry, tidal wetting and drying, and irregular coastlines^27,34–36^. In this work, considering the computational demand for continuous long-term simulations, we chose the 3D barotropic and hydrostatic version, which resolves simplified Reynolds-averaged Navier-Stokes equations (with Boussinesq approximations), where an external mode solves 2D depth-integrated equations and the internal mode solves the equations in three dimensions.

The model domain extends ~700 km offshore from the mid-Atlantic coast to adequately capture the air-sea interaction during hurricane landfalls (Fig. 3) and ~215 km from the Delaware Bay mouth to the river flow boundary (Fig. 1c). It also extends landward on the eastern and western shores of DBR, up to a floodplain boundary of 3 m elevation above the North American Vertical Datum of 1988 (NAVD88), to correctly resolve the inundation of low-lying Delaware and New Jersey floodplains. We collect the topographic and bathymetric data from a combination of National Oceanic and Atmospheric Administration (NOAA) DEM sources such as the 1-arc-minute Earth TOPOgraphy (ETOPO1) and 1/9-arc-seconds Coastal Relief Models, and use the Surface Water Modeling software (SMS) version 13.0 to generate the model grid. The pre-defined size function assigned for different parts of the study area (e.g., tidal river, bay, and open ocean) provided a varying horizontal resolution from ~30 km along the open ocean boundary to 30 m in the upstream and narrowest portion of the Delaware River, providing a reasonable number of 219k triangle elements and 116.5k nodes considering the period of model simulation. To represent the tidal forcing and sea-level trend at the open boundary, we used the ADvanced CIRCulation (ADCIRC) model ec2001 tidal database^37,38^ and the observed historical sea-level trend from NOAA’s Center for Satellite Applications and Research (STAR) repository^39^. They are combined to provide the total water level at the boundary, which is necessary for taking care of the temporal and spatial evolution of the sea level within our study area. Then, from the hundreds of DHSVM streams with outlets near the Delaware River, we picked 13 major streams with a 99.5th percentile of peak flow above 100 m3/s, which significantly contributed to overall flooding during extreme events. Limiting the number of narrow streams that require much higher resolution helped increase the model external time-stepping – a critical factor in this work due to the long simulation period. With the assigned model setup and configuration, the continuous 40-year model run required ~1.1 × 10^6^ CPU hours with Intel Xeon Haswell processor nodes in the National Energy Research Scientific Computing Center (NERSC) CORI supercomputing system. We used four nodes, each consisting of 64 processors (a total of 256 processors).

### Tidal analysis and Floodwater Depth estimate

**Fig. 4:**
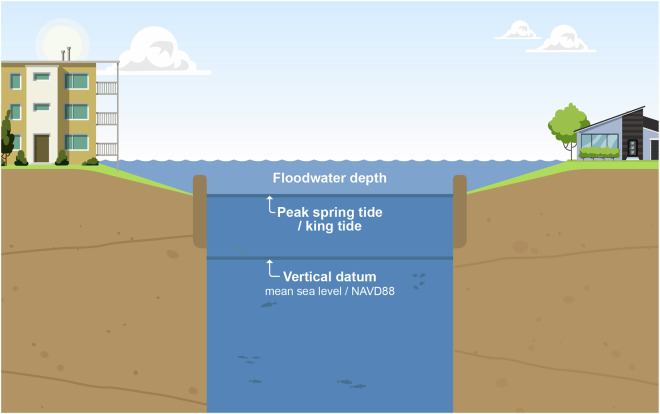
Sketch showing the peak spring tide/king tide and Floodwater Depth estimates from the North American Vertical Datum of 1988 (NAVD88) in estuarine channels. We estimate this ‘Floodwater Depth’ by subtracting the yearly peak tidal elevation (king tide) from the total water level.

In estuaries experiencing semi-diurnal and spring/neap tides, identifying flood events caused by storm surges can be challenging due to the timing of surges relative to the tidal stage. Many studies have directly used the time series of total water level (TWL) from a specific datum to represent surge-driven flooding. Some have used non-tidal residual^40^ (NTR; the difference between TWL and predicted tide), while others used skew surge^41^ (a measure of the difference between the maximum TWL and the maximum predicted tidal level during a tidal cycle), allowing an offset of recorded and predicted tidal peaks (i.e., skewed) from each other. While both these terms, NTR and skew surge, estimate the impact of meteorologically forced surges on water level during extreme events, they might not always represent flooding in a tidal estuary or river. In the existing literature, hourly NTR is a standard measure of storm surge that includes non-linear interactions between tide, river discharge, and low-frequency surge^42,43^. However, NTR, as a proxy for flooding, could have higher uncertainties as the maximum NTR might not always occur during the predicted peak high tide. A large NTR occurring during low/ebb tide, compared to a small NTR during high tide, does not necessarily indicate more severe flooding. Recent studies have utilized skew surge, which is uninfluenced by the tidal phase but measures the difference between the predicted and recorded water level peaks during the same tide cycle^19^. Even with the additional adjustments, the skew surge might mislead the estimate of flooding during spring-neap variations in tidal estuaries. For example, a higher skew surge during a neap tide cycle would not necessarily mean the exact extent of overland flooding compared to a spring cycle. Thus, in this work, we identify flooding or flood events by measuring a difference between the TWL and peak tide (hereafter called ‘Floodwater Depth’) where we estimate the peak tidal elevation over a much more extended period of 1 year (shown in Fig. 4).

The Floodwater Depth provides a straightforward representation of coastal flooding by assuming that the yearly peak tidal elevation (king tide) does not represent flood, and the water level surpasses that elevation can potentially represent a flood depth that also overtop the river banks. This estimate is also better than directly using the TWL for EVA models as it allows us to differentiate between the peak tidal elevation and the storm-induced flooding, which is essential for long-term planning and management. It also eliminates the threshold selection for the extreme value analysis using POT/GPD analysis, which is critical for return level estimates. Here, by default, the spatially varying peak tidal elevation becomes the threshold choice of the high water from which we assume that the overland flood begins. We want to note that while our Floodwater Depth metric represents a storm-induced water depth along the bay and river, it is not equivalent to the flood warning threshold used by the National Weather Service for hazard and impact assessments^44^, which is based on the depth of inundation over land.

**Fig. 5:**
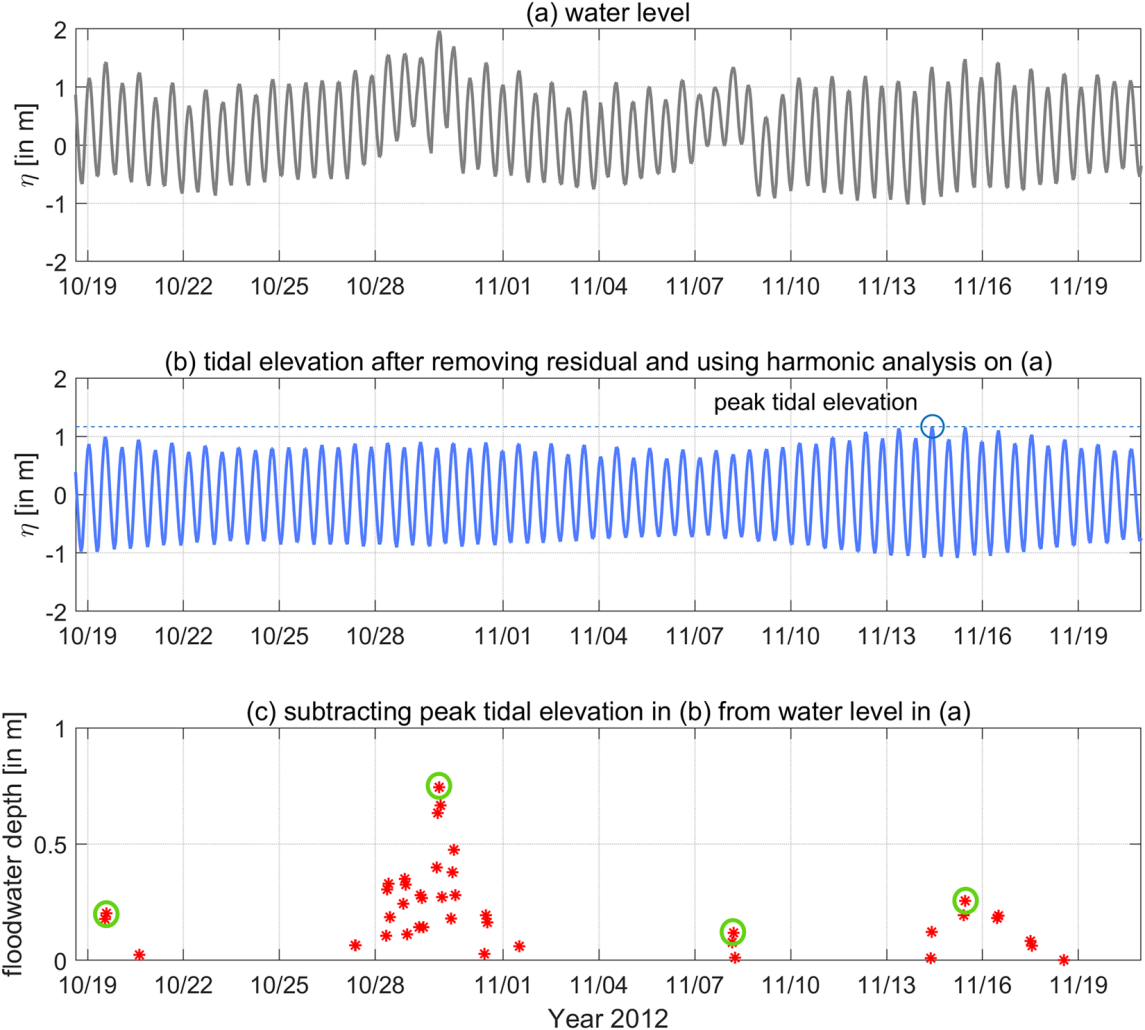
Example plot showing the estimation of Floodwater Depth from the total water level time series data. (**a**) Time series of Total Water Level (TWL) at Ship John Shoal, NJ, NOAA tide gauge; (**b**) Pure tidal oscillation after removing the long-term trend and subtidal signals from TWL and using harmonic analysis. The dashed line represents the peak tidal elevation; (**c**) Time series of Floodwater Depth after removing the peak tidal elevation from TWL. Here, the green circles represent the peak Floodwater Depth events selected based on the POT/GPD threshold of 0.05 m and a declustering time of 3 days for event independence.

The process of estimating the Floodwater Depth is shown in Fig. 5 where we separate the TWL signal into tidal and non-tidal parts, estimate the annual peak tidal elevation considering the sea level trend, and finally extract the time series of the Floodwater Depth. We first applied this method to the observation datasets collected from the NOAA Tides and Currents database (https://tidesandcurrents.noaa.gov/). Within our study area, ten tide gauge locations have historical water level datasets of different periods and are currently in service. However, only six gauge locations, Lewes (LW), Brandywine Shoal Light (BSL), Ship John Shoal (SJS), Reedy Point (RP), Philadelphia (PHL), and Newbold (NB), have measurements for an extended period and represent different regions of the estuary – progressively going from the bay mouth to the upstream river (Fig. 1c). They also have different time resolutions, such as 6-min intervals, hourly, and high/low per tide cycle (six-hourly). For this study, first we collected hourly data for 2011 from the six gauges that had continuous measurements without any missing periods to do the tidal analysis. We used this hourly record for harmonic analysis and finding peak tidal elevations. Later, we compared these datasets with model results for the same period and provided an estimate of the model bias in predicting peak tidal elevations. Before continuing with model results for extreme value analysis and return level estimates, we verified model performance in predicting the isolated extreme event TWL. When looking at the most important events for the study region^41^, all of our gauge locations consisted of hourly TWL data for Hurricane Isabel (2003), Wilma (2005), Ernesto (2006), Irene (2011), and Sandy (2012). While it is possible to obtain TWL data for additional historical events, most gauges record six-hourly data with irregular gaps, and have less than 40 years of records, except for LW and RP. This gap also prevented us from calculating return levels at most field locations except RP, limiting our evaluation of the numerical model’s performance for statistical estimates to only the RP tide gauge.

Before using the TWL data for any statistical analysis, the dataset was detrended to make it independent and stationary to represent the random process. This step is crucial for extreme value analysis, where the assumption is that the distribution of any sample subset remains the same^17,31^. However, in coastal or estuarine systems, TWL time series can contain significant trends due to the increase in the sea level over time or periodicity from the seasonal or long-term oscillations of the open ocean. It is thus necessary to remove the trend or periodicity from the signal to ensure that the mean and standard deviation of the process remain unchanged over time, thereby maintaining the assumption of stationarity. In this study, we first implemented a tide/wave averaging approach that removes the long-term trend and subtidal signals from TWL. Then, we conducted a harmonic analysis using tidal analysis software, ‘t-tide,’ to get the purely tidal oscillation for each year (tool link is available in the Code availability section). To get the peak tidal elevation for any part of the estuary, we superimposed the long-term trend with the tidal signal and located the annual peak elevations over 40 years. We used Matlab’s ‘detrend’ function to get the signal trend, which performs a linear fit to the time-series data and separates the trend (for more details see the link provided in the Code availability section). This analysis gave us the peak tidal elevations for the entire study region, which we then used to calculate the Floodwater Depth. Finally, combining the peak tidal elevation and the Floodwater Depth magnitude for different recurrence intervals, we generated the stillwater elevation dataset - commonly used by FEMA for flood risk mapping and assessment.

### Extreme value analysis and Floodwater Depth Return Levels

Extreme Value Analysis (EVA) is a statistical field that explores and models the likelihood of rare and severe events, such as extreme rainfall, storm surges, and wave heights. These events usually occur at the extreme ends—so called “tails"—of probability distributions and can have significant consequences in hydrology, coastal science, and climate science. Various statistical distributions are used to analyze extreme values within a time series, including Generalized Extreme Value (GEV), encompassing the Extreme Value Type I (Gumbel), Type II (Fréchet), and Type III (Reverse Weibull) distributions, the Pearson and Log-Pearson Type III, Generalized Pareto Distribution (GPD), and Lognormal Distributions (for an overview, see Merz *et al*.^45^). These time series may represent either a sequence of maximum values occurring within equal time intervals (such as months or years) or encompass all values exceeding a specified extreme threshold. The process involves fitting different analytical functions to the data to select the most suitable function to represent the distribution of extreme values. In coastal studies focusing on US coastlines, many methods have been applied to perform EVA on TWL^46–50^. Among them, GEV and GPD have been most frequently applied to estimate return levels and periods of extreme water levels from coastal ocean datasets.

The GEV distribution is commonly used to fit the maximum values of non-overlapping blocks within a time series when the events are assumed to be independent. This method is called the Block Maxima (BM) approach and is often used to characterize annual maximum values. On the other hand, the GPD is used to fit the upper tail of the parent distribution. This tail comprises values exceeding a certain threshold, referred to as the Peaks-Over-Threshold (POT) or Partial Duration Series method^51,52^. Like the BM method, the POT method assumes the events are independent. Although POT provides a more straightforward representation of extreme values than BM, it is essential to note that the temporal clustering of data points and the subjective selection of a threshold can introduce complexities in the analysis^31^. While both the GEV and GPD distributions are characterized by three parameters—location, scale, and shape—the GPD uses a threshold value in place of the location parameter. This threshold is not an additional parameter but rather specifies the minimum value for which the GPD is applicable, essentially defining the starting point of the tail distribution that the GPD models.

The cumulative distribution function (CDF) of the GEV distribution consists of three families, and they can be written as:

For *ξ* = 0 (Gumbel):

1 $$F(x;\mu ,\sigma )=\exp \{-\exp [-\frac{(x\;-\;\mu )}{\sigma }]\}$$

For *ξ* < 0 (Weibull) and *ξ* > 0 (Fréchet):

2 $$F(x;\mu ,\sigma ,\xi )=\exp \{-{[1+\frac{\xi (x-\mu )}{\sigma }]}^{-\frac{1}{\xi }}\}$$

where *μ*, *σ*, and *ξ* are the location, scale, and shape parameters, respectively. The Weibull distribution has a lower chance of including a value significantly greater than *μ*. The Gumbel distribution is considered a light-tailed distribution, while the Fréchet distribution is known as a heavy-tailed distribution because it is more likely to include a value much larger than *μ*.

The CDF of GPD, the second approach, can be defined as:

For *ξ* ≠ 0 :

3 $$F(x;\mu ,\sigma ,\xi )=1\;-\;{[1+\frac{\xi (x-\mu )}{\sigma }]}^{\frac{1}{\xi }}$$

For *ξ* = 0 :

4 $$F(x;\mu ,\sigma )=1\;-\;\exp (-\frac{x\;-\;\mu }{\sigma })$$

where *μ* is the threshold, *σ* is the threshold-dependent scale parameter (>0), and *ξ* is the shape parameter.

In EVA, various methods are commonly employed to estimate scale and shape parameters, such as Maximum Likelihood Estimation (MLE), Method of Moments, L-moments^53^, and Probability-Weighted Moments (PWM)^54^. MLE is often preferred for its general applicability and efficiency in estimating the unknown parameters of a distribution^31^. Subsequently, the confidence intervals for the estimated parameters can be calculated using the bootstrap Monte Carlo method. In this work, we used ‘pyextremes’ to efficiently perform EVA with large datasets. This toolbox has options for GEV, GPD, and the MLE method to fit the models. To get the extreme Floodwater Depth, we used the annual maxima for BM/GEV, and for POT/GPD, we assumed a declustering time of 3 days for event independence.

## Data Records

**Fig. 6:**
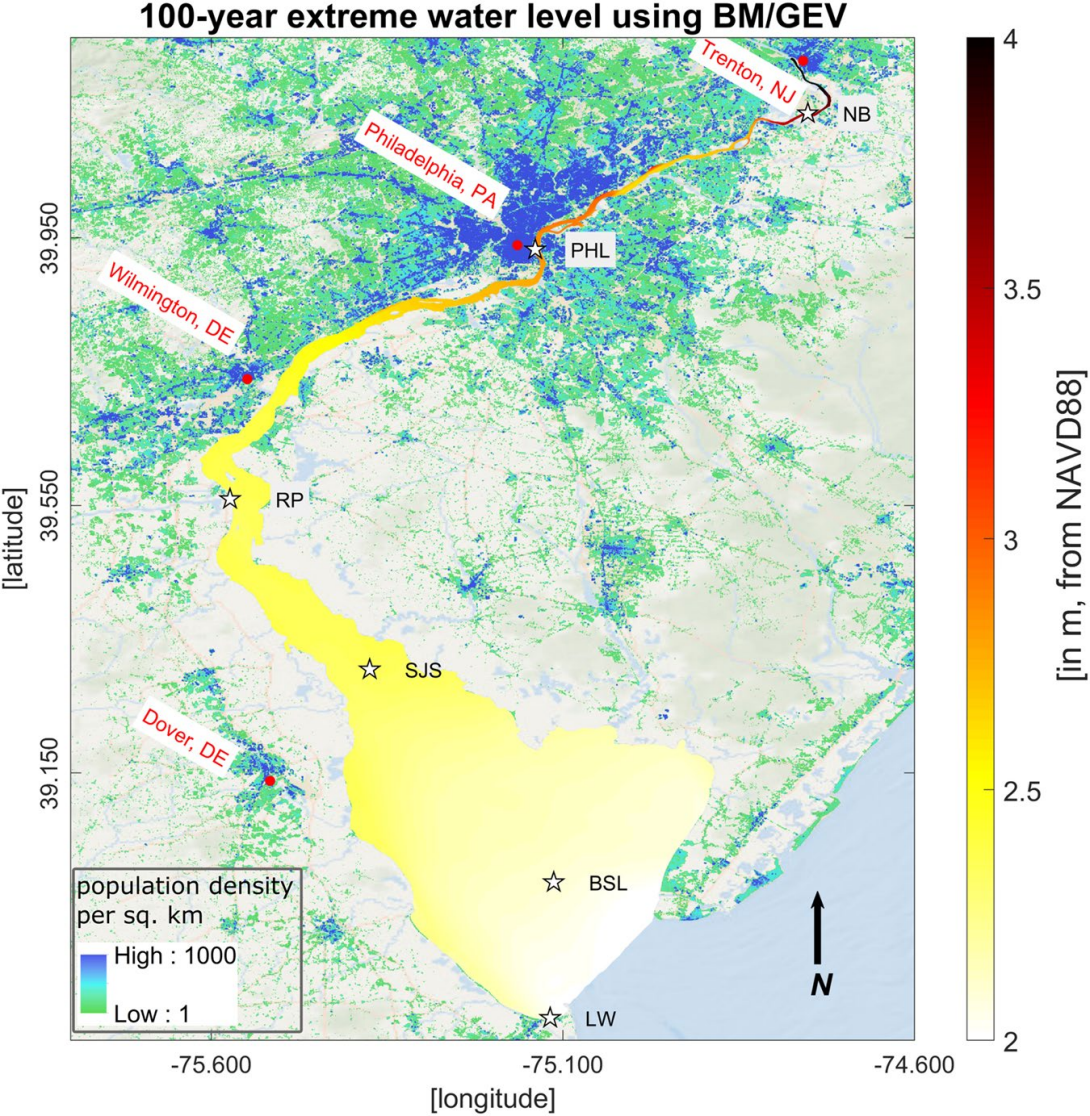
2D map of the 100-year extreme flood return level using BM/GEV. Here, this return level represents the stillwater elevation (in meters, from NAVD88) by combining the 100-year flood water depth and peak tidal elevation at model grid nodes that cover the bay/channel water body. The LandScan USA (2021) population density data (1-km resolution) is added on top of the background map.

**Fig. 7:**
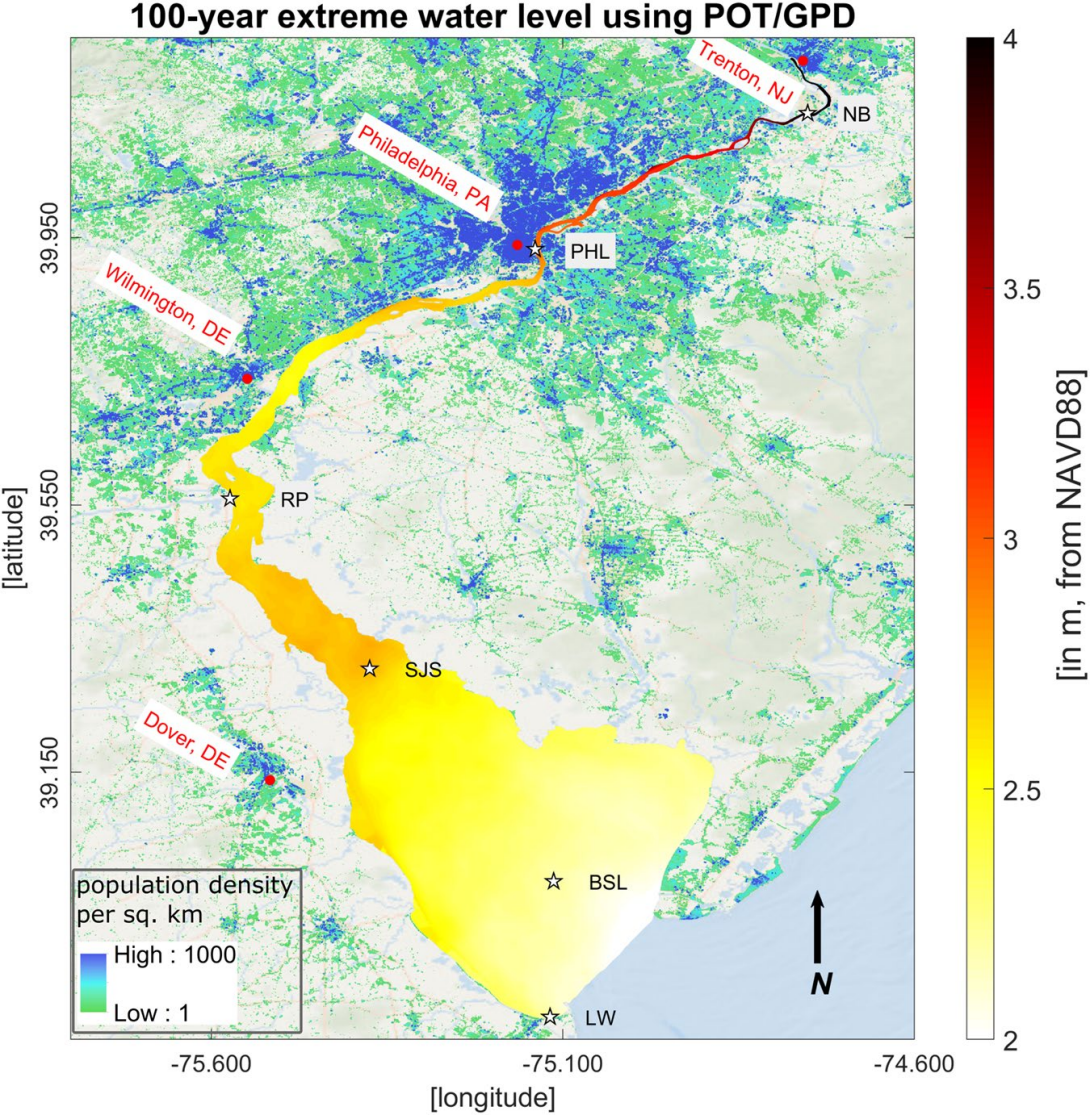
2D map of the 100-year extreme flood return level using POT/GPD. The return level represents the stillwater elevation (in meters, from NAVD88) combining the 100-year flood water depth and peak tidal elevation at model grid nodes that cover the bay/channel water body. The LandScan USA (2021) population density data (1-km resolution) is added on top of the background map.

**Fig. 8:**
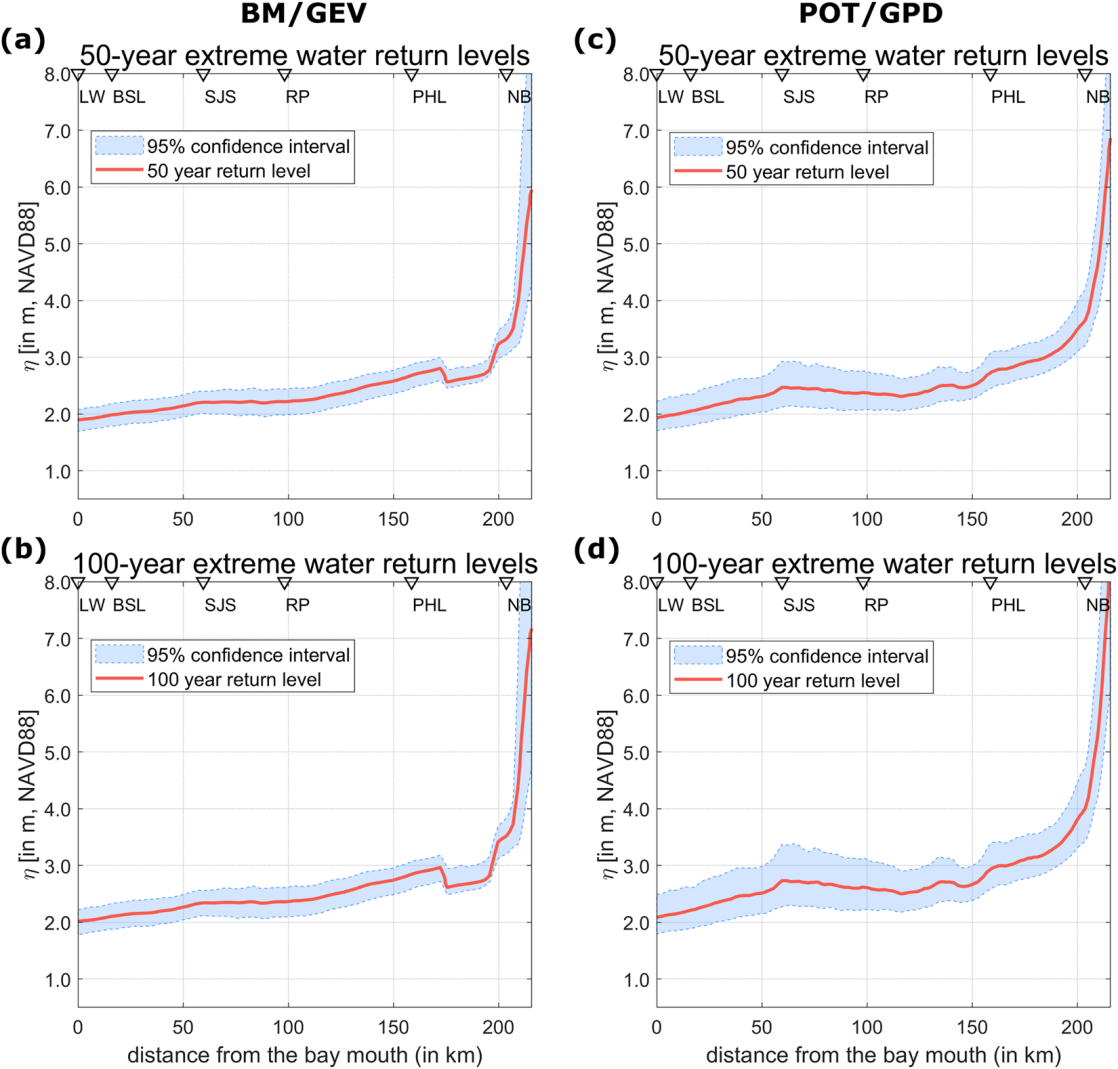
(**a,c**) Along-channel (from bay entrance to the upstream model river boundary) 50-year stillwater elevation return levels with 95% confidence interval for both BM/GEV and POT/GPD; (**b,d**) Along-channel 100-year stillwater elevation return levels with 95% confidence interval for both BM/GEV and POT/GPD.

The Floodwater Depth and stillwater elevation return levels are accessible to the public via the MSDlive repository: 10.57931/2350826^55^. Table 1 describes all the files representative of flood processes and those used for model evaluation, including their structure and dimensions. The large files are mainly kept in MatLab (.mat) formats, and essential return level values are given in Microsoft Excel (.xslx) formats for easier access. The raw water surface elevation data from the model is also provided, allowing user to estimate return levels from scratch. Using these datasets, users can produce stillwater elevation maps for different return periods, as shown in Figs. 6 and 7 and the along-channel return level variations shown in Fig. 8. Figures 6 and 7 illustrate how the return levels vary spatially along the channel, showing greater amplification near PHL and in the section of the river extending to Trenton, NJ. In these plots, we also added the 1-km resolution LandScan USA (2021) population density data (https://landscan.ornl.gov/) on top of the background map to illustrate where the Floodwater Depth may have a greater impact in the region. More discussion about the non-linear interaction between tide, surge, river discharge, and the complex geometry and their effects on flood amplification and dampening is given in Deb *et al*.^27^.

**Table 1:** Description of the extreme flood dataset developed for the DBR region.

File Name	Naming Convention	Data File Description
WSE-modelVSfield-table.mat	Observation and model water surface elevation (in meters, from NAVD88) from six NOAA tide gauge locations used for model validation during major storm events that impacted DBR. This data is used in generating statistics in Table 2.	
	**matlab (.mat) file**	WSE_Lon_Lat [dim: station names x longitude/latitude]; WSE_time [character strings of date-time for different events and locations]; WSE_model [water surface elevation from model; WSE_field [water surface elevation from field]
WSE-DE-Bay-Full.mat	Water level/surface elevation time-series (in meters) at 1681 point locations from 1980-2019 based on the FVCOM model simulation. This surface represents the water level variation from the NAVD88 vertical datum.	
	**matlab (.mat) file**	WSE_DE_Bay.time [array of date-time strings]; WSE_DE_Bay.WSE [Water level/surface elevation time-series]; WSE_DE_Bay.longitude/latitude [point coordinates in the North American 1983 datum (NAD83)]
Peak-Tidal-Elevation.mat	Annual peak tidal elevation at 1681 point locations from 1980-2019 based on FVCOM model data. We removed the long-term trend and subtidal signals from the total water level, conducted a harmonic analysis to get the purely tidal oscillation for each year, and then superimposed the long-term trend with the tidal signal to locate this peak elevation.	
	**matlab (.mat) file**	peak_tidal.tidal_WSE [tidal elevation, dimension: point locations x years (1980-2019)]; peak_tidal.longitude/latitude [point coordinates]
field-model-RL-comparisons.xlsx	Floodwater depth return value difference (in meters) between model and field at a NOAA tide gauge location, Reedy point, DE, along with 95% confidence intervals using BM/GEV and POT/GPD. Part of this data is provided in Table 4.	
	**microsoft excel (.xlsx) file**	data dimension: return period x return level x lower 95% confidence interval x higher 95% confidence interval
model-GEV-GPD-RL-comparisons.xslx	Floodwater depth return value estimates using Extreme Value Analyis methods, BM/GEV and POT/GPD, along with 95% confidence intervals at different regions of the study area: upstream river (Newbold, PA) and bay (Ship John Shoal, NJ).	
	**microsoft excel (.xslx) file**	data dimension: return period x return level x lower 95% confidence interval x higher 95% confidence interval
FWD-DE-Bay-Full.mat	Floodwater depth time-series (in meters) at 1681 point locations from 1980-2019. Here, the yearly peak tidal elevation is removed from the continous water surface elevation.	
	**matlab (.mat) file**	FWD_DE_Bay_Full.time [array of date-time strings]; FWD_DE_Bay_Full.floodwater_depth [floodwater depth time-series]; FWD_DE_Bay_Full.longitude/latitude [point coordinates]
Floodwater-Depth-RL-GEV.xlsx	Floodwater depth return levels at 1681 point locations using BM/GEV for 10, 25, 50, and 100-year period extreme events (in meters).	
	**microsoft excel (.xslx) file**	the excel file has four sheets representing different return periods; data dimension: longitude x latitude x return level x lower 95% confidence interval x higher 95% confidence interval
Floodwater-Depth-RL-GPD.xlsx	Floodwater depth return levels at 1681 point locations using POT/GPD for 10, 25, 50, and 100-year period extreme events (in meters).	
	**microsoft excel (.xslx) file**	dimension is same as the previous file
Stillwater-RL-GEV.xslx	Extreme water return levels at 1681 point locations using BM/GEV by merging peak tidal elevation of year 2019 with the Floodwater Depth return levels. This data is shown in Figs. 6 and 8a,c.	
	**microsoft excel (.xslx) file**	data dimension: longitude x latitude x return levels
Stillwater-RL-GPD.xslx	Extreme water return levels at 1681 point locations using POT/GPD by merging peak tidal elevation of year 2019 with the Floodwater Depth return levels. This data is shown in Figs. 7 and 8b,d.	
	**microsoft excel (.xslx) file**	data dimension: longitude x latitude x return levels

## Technical Validation

### Hydrodynamic model performance

To ensure the technical quality and reliability of the dataset, we performed rigorous model comparisons with the available observation data for both DHSVM and FVCOM models. The DHSVM simulations of river discharge within the Delaware River basin have been thoroughly evaluated in prior studies, as documented by Sun *et al*.^56^ and Cooper *et al*.^57^. For example, the Kling-Gupta Efficiency (KGE; Equation (5)) for daily flows, when compared against long-term (mostly over 30 years) observations collected from six United States Geological Survey (USGS) along the Delaware mainstem, ranged from 0.6 to 0.73; a KGE of 1 indicates perfect prediction. Despite the favorable KGE scores, the DHSVM simulations introduce biases that are propagated into the FVCOM TWL simulations through the coupling process.

5 $$KGE=1\,-\,\sqrt{{(R-1)}^{2}+{(\frac{{\sigma }_{m}}{{\sigma }_{o}}-1)}^{2}+{(\frac{{\mu }_{m}}{{\mu }_{o}}-1)}^{2}}$$

Where R is the linear correlation coefficient, *σ*_*o*_ is the standard deviation in observations, *σ*_*m*_ is the standard deviation in simulations, *μ*_*m*_ is the simulation mean, and *μ*_*o*_ is the observation mean.

**Fig. 9:**
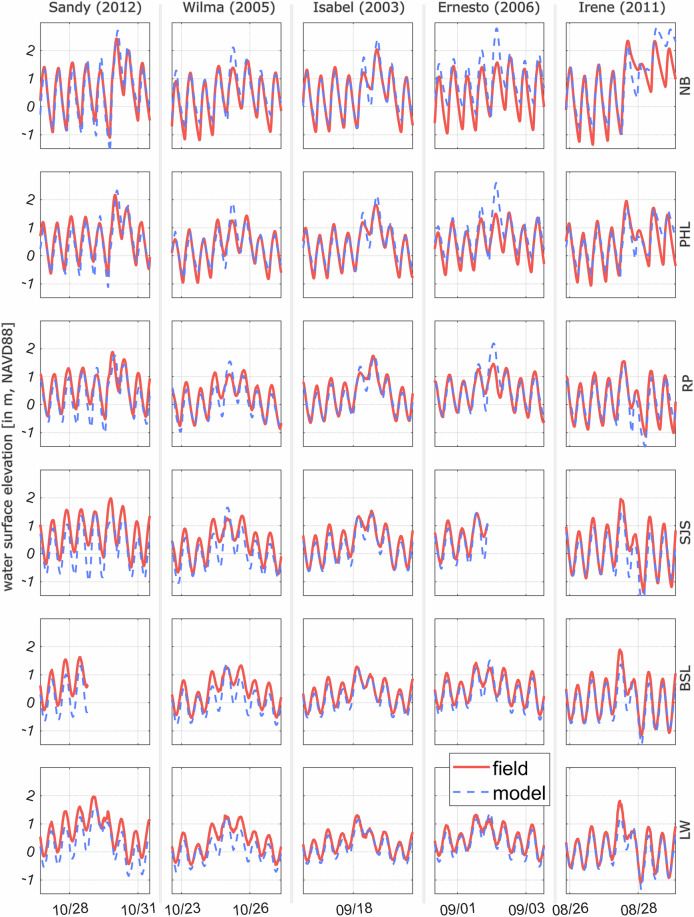
Comparison of model and observed water levels during five major storm events at different NOAA tide gauge locations. The vertical subplots, arranged from top to bottom, illustrate a gradual change in the properties of the tidal systems, transitioning from river portion (Newbold, NJ) to the bay entrance (Lewes, DE).

As this study mainly focuses on predicting the extreme water level in the estuary and river, we prioritized to address the biases or errors in the FVCOM-generated water level dataset. We separated the model assessment into three parts that can progressively and systematically display the error generation. At first, we compared TWL at various NOAA gauge locations during extreme events that extensively affected the area and during which the gauges continuously recorded the TWL. Figure 9 shows the comparison between the field and model during five extreme events: Hurricane Sandy (2012), Wilma (2005), Isabel (2003), Ernesto (2006), and Irene (2011), which had the most impact in the study area among various other events. In this plot, different parts of the estuary are represented by the location of the subplot (e.g., rows), starting from Delaware Bay (LW) at the bottom and ending at the upstream portion of the Delaware River (NB). DBR is a complex, shallow, and convergent system where the tidal amplitude initially reduces due to frictional dissipation and then slowly increases again as the estuary width narrows with distance and the incident wave interacts with the reflected wave in the tidal river (More discussion provided in Deb *et al*.^27^). This phenomenon can be seen in Fig. 9; as the storm wave propagates upstream, the peak of water surface elevation increases with distance. In the river-estuary interaction zone (NB to RP), river discharge from extreme event precipitation also elevates the sea level while damping the tidal amplitude [water level at NB during Hurricane Irene (2011) shown in Fig. 9]. Spatial variations in biases from atmospheric and river forcing inputs, combined with spatially varying hydrodynamic processes, lead to spatial differences in model performance in predicting TWL. We calculated three commonly used model performance metrics (Table 2) in the coastal modeling community: *Correlation coefficient*, *Averaged bias index*, and *Skill* (equations (6)–(8)), using WSE records from six tide gauge locations during five extreme events. The metrics, averaged across all events for each gauge, are detailed in Table 1. We can see that the *Correlation coefficient* and *Skill* range from ~0.91–0.97 and ~0.91–0.94, respectively. These values indicate strong agreement with observations, where 1.0 represents complete agreement. However, the *Averaged bias index* shows that the model underpredicts WSE progressively from upstream/river (NB) to downstream/bay entrance (LW) location, where the maximum difference reaches ~0.18 m.

**Table 2:** FVCOM model performance statistics at the six tide gauge locations using water level during five extreme events shown in Fig. 9.

Location/Metrics	Avg. bias	Skill	Corr (R)
Newbold (NB)	0.075	0.929	0.917
Philadelphia (PHL)	−0.002	0.936	0.907
Reedy Point (RP)	−0.071	0.941	0.919
Ship John Shoal (SJS)	−0.154	0.906	0.937
Brandywine Shoal Light (BSL)	−0.147	0.933	0.964
Lewes (LW)	−0.183	0.919	0.965

All the metrics are averaged over five events. A positive bias represents model over-prediction, while a negative bias represents under-prediction.

As mentioned earlier in section Tidal analysis and Floodwater Depth estimate 0.0.2, in this work, we are separating the WSE into two parts: the peak tidal elevation and Floodwater Depth. Hence, comparing the model-predicted tidal constituent amplitude with observation is essential before analyzing the return levels of Floodwater Depth. Tidal constituents are the individual harmonic components that collectively define the tidal signal, each corresponding to a specific astronomical or gravitational cycle. For example, the M2 constituent represents the principal lunar semidiurnal tide, while S2 reflects the solar semidiurnal influence. In Table 3, we provided the FVCOM model-predicted amplitude of the major tidal constituents and their difference with the field data at the tide gauges in DBR, using the continuous yearly dataset of 2011 (as mentioned earier, the year 2011 is chosen due to data availablity at all gauges). The semi-diurnal constituents (M2, N2, and S2) primarily dominate this region, and we observe that the principal lunar semidiurnal constituent (M2) has the largest amplitude in all of the gauges. The amplitude error gradually increases from LW to NB, where the model underpredicts the M2 amplitude by ~0.19 m at NB. To achieve higher accuracy in the river-dominated portion of DBR, it is essential to represent small channels, tributaries, and floodplains with higher resolution, which is challenging considering the computational expense required for a long-term 40-year simulation. Based on this assessment, we suggest that users of this dataset take into account these errors when interpreting the data.

6 $$Correlation\,coefficient,R=\frac{{\sum }_{n=1}^{N}({M}_{n}\,-\,\overline{M})({O}_{n}\,-\,\overline{O})}{\sqrt{({\sum }_{n=1}^{N}{({O}_{n}-\overline{O})}^{2})({\sum }_{n=1}^{N}{({M}_{n}-\overline{M})}^{2})}}$$

Where *M*_*n*_ and *O*_*n*_ are simulated and observed water levels, *N* is the total number of observations used in metric calculations, and $\overline{O}$ is the mean of observed sample *N*.

7 $$Avg.bias\,index=\frac{{\sum }_{n=1}^{N}({M}_{n}\,-\,{O}_{n})}{{\sum }_{n=1}^{N}{O}_{n}}$$

8 $$Skill=1\;-\;\frac{{\sum }_{n=1}^{N}{({M}_{n}-{O}_{n})}^{2}}{{\sum }_{n=1}^{N}{(| {M}_{n}-\overline{O}| +| {O}_{n}-\overline{O}| )}^{2}}$$

**Table 3:** FVCOM model error in predicting the tidal amplitude using a full year (2011) simulation and observation data at six tide gauge locations (in meters).

tidal constituents	amplitude (in m)	NB	PHL	RP	SJS	BSL	LW
M2	*a*_*obs*_	1.053	0.829	0.785	0.839	0.721	0.591
	*a*_*mod*_	0.864	0.647	0.639	0.726	0.686	0.594
	*a*_*dif*_	−**0.189**	−**0.182**	−**0.146**	−**0.112**	−**0.034**	**0.002**
N2	*a*_*obs*_	0.180	0.144	0.148	0.169	0.160	0.138
	*a*_*mod*_	0.137	0.105	0.116	0.139	0.141	0.127
	*a*_*dif*_	−**0.042**	−**0.039**	−**0.032**	−**0.030**	−**0.018**	−**0.011**
S2	*a*_*obs*_	0.116	0.091	0.102	0.123	0.123	0.107
	*a*_*mod*_	0.120	0.091	0.105	0.127	0.127	0.113
	*a*_*dif*_	**0.004**	**0.001**	**0.003**	**0.004**	**0.004**	**0.006**
K1	*a*_*obs*_	0.089	0.074	0.068	0.074	0.068	0.063
	*a*_*mod*_	0.062	0.053	0.053	0.054	0.050	0.047
	*a*_*dif*_	−**0.027**	−**0.021**	−**0.014**	−**0.020**	−**0.018**	−**0.016**

Here, we only show the major semi-diurnal and diurnal tidal constituents M2, N2, S2, and K1. The difference between the amplitudes is estimated by subtracting observation from the model.

### Verification of the parametric (EVA) models

**Fig. 10:**
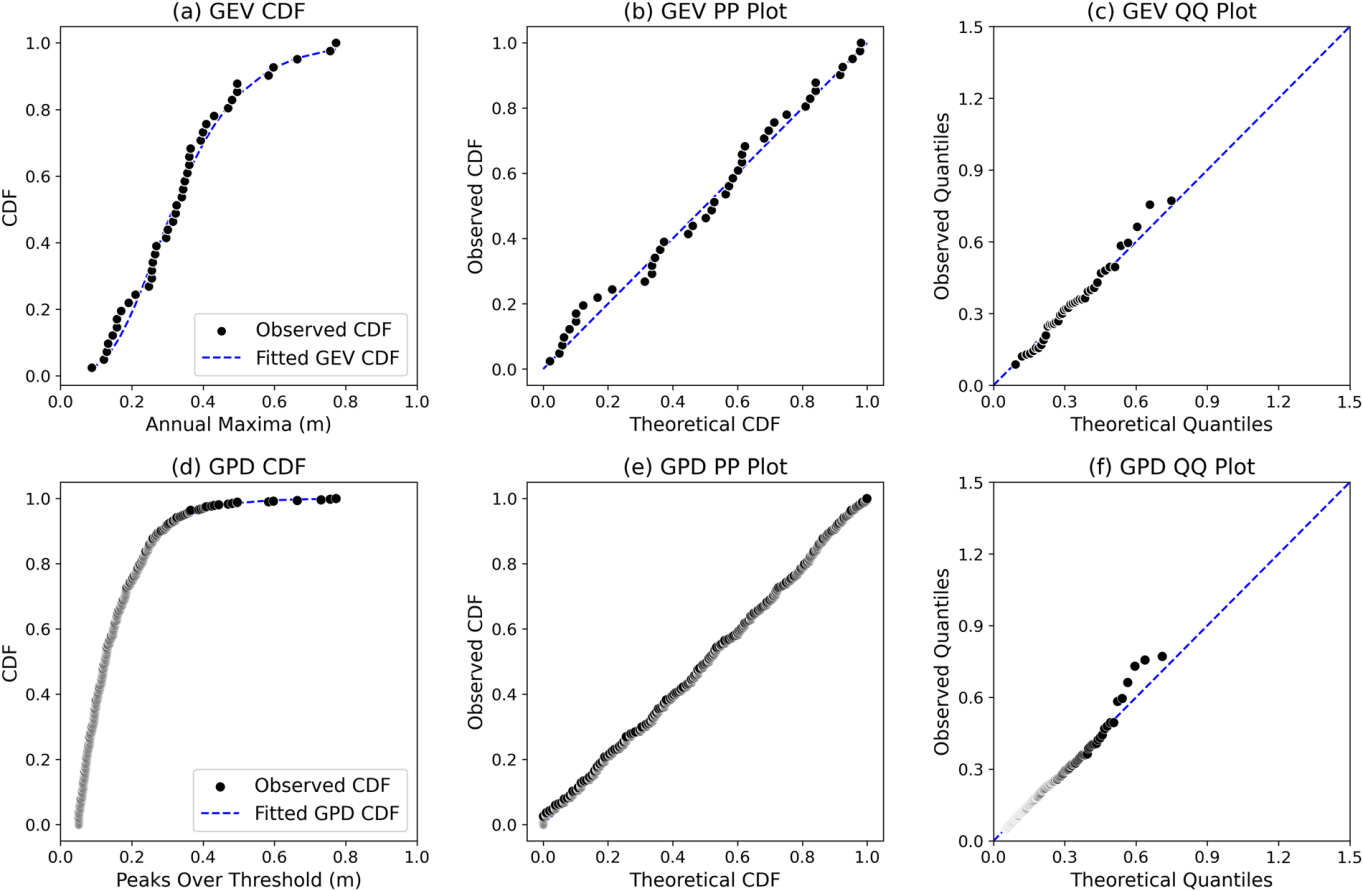
(**a**) Comparison of cumulative distribution functions (CDFs) derived from empirical (observed) data and Generalized Extreme Value (GEV) estimates, based on the field gauge annual maxima of Floodwater Depth; (**b**) Probability-Probability (P-P) plot comparing the cumulative probability of empirical data with the fitted GEV distribution; (**c**) Quantile-Quantile (Q-Q) plot comparing the quantiles of empirical data with those from the fitted GEV distribution; (**d,e,f**) Corresponding CDF, P-P, and Q-Q plot comparisons for the Peaks over Threshold (POT) and Generalized Pareto Distribution (GPD).

**Fig. 11:**
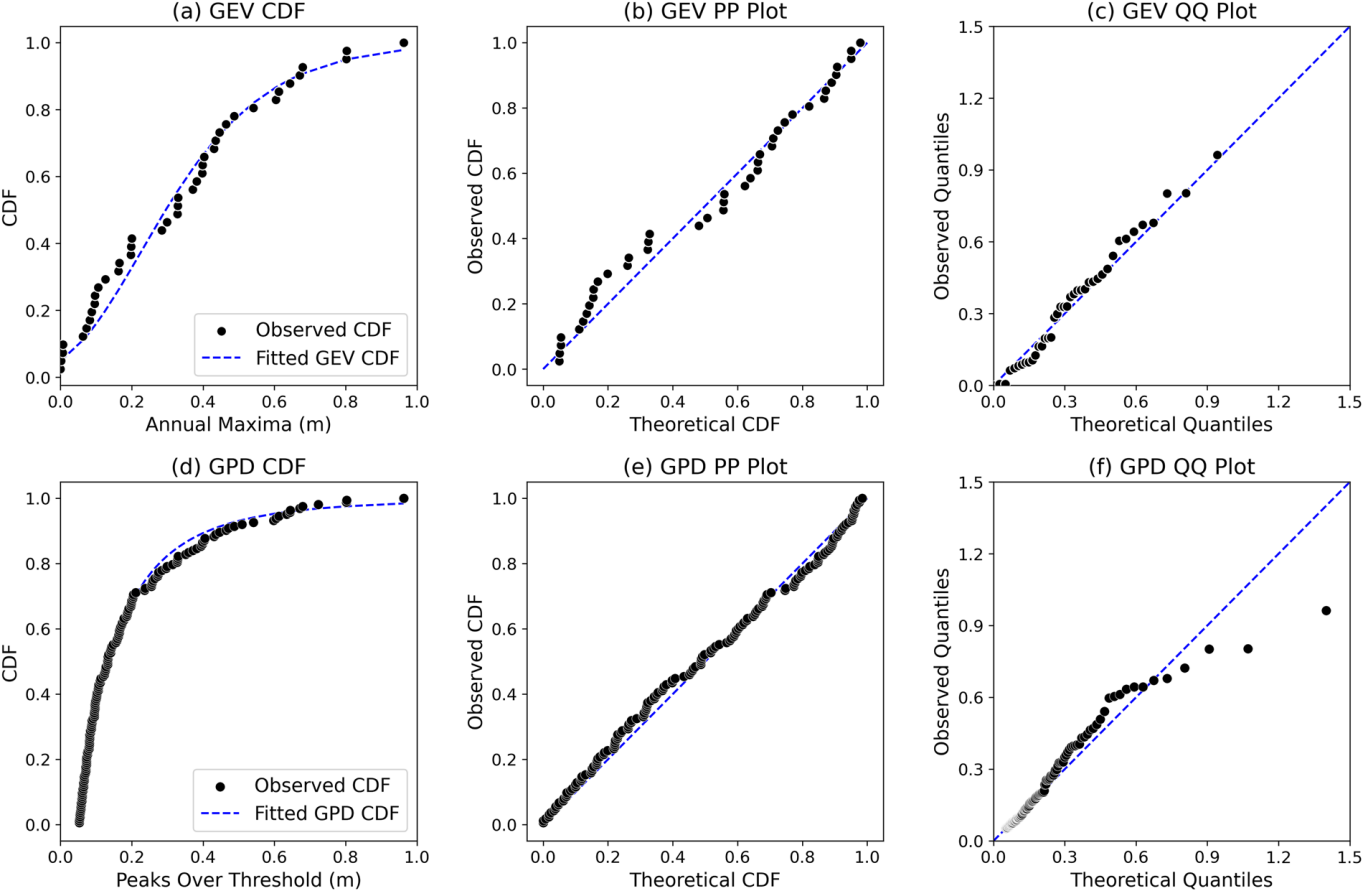
(**a**) Comparison of cumulative distribution functions (CDFs) derived from empirical (observed) data and Generalized Extreme Value (GEV) estimates, based on the FVCOM model-generated Floodwater Depth data; (**b**) Probability-Probability (P-P) plot comparing the cumulative probability of empirical data with the fitted GEV distribution; (**c**) Quantile-Quantile (Q-Q) plot comparing the quantiles of empirical data with those from the fitted GEV distribution; (**d, e, f**) Corresponding CDF, P-P, and Q-Q plot comparisons for the Peaks over Threshold (POT) and Generalized Pareto Distribution (GPD).

**Fig. 12:**
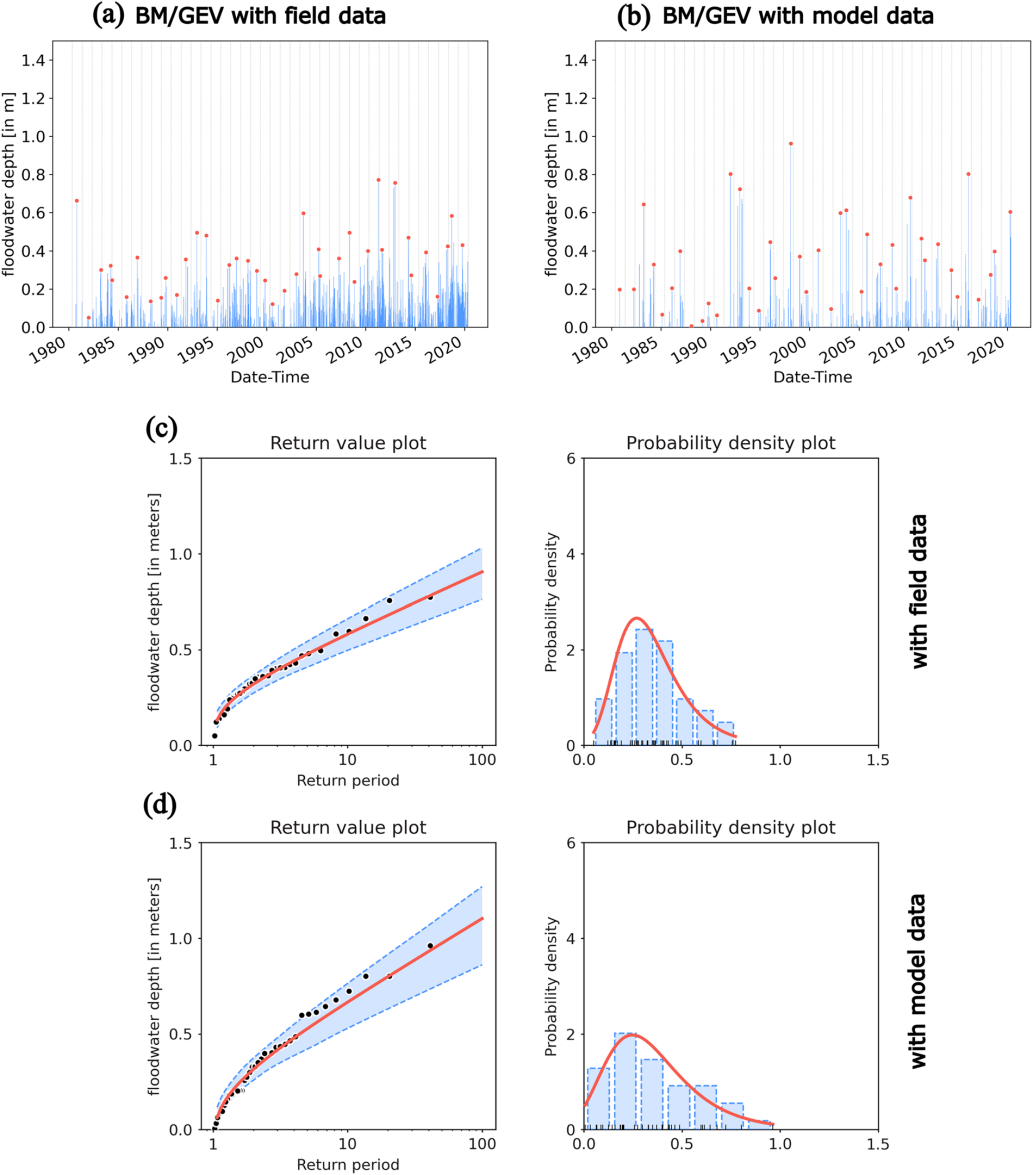
(**a**) Annual maxima of Floodwater Depth from 40-year period field dataset at Reedy Point, DE; (**b**) Annual maxima of Floodwater Depth from 40-year period model dataset at Reedy Point, DE;; (**c**) return value estimate and probability distribution function (PDF) of the Floodwater Depth using BM/GEV based on field data; (**d**) return value estimate and PDF of the Floodwater Depth using BM/GEV based on model data.

**Fig. 13:**
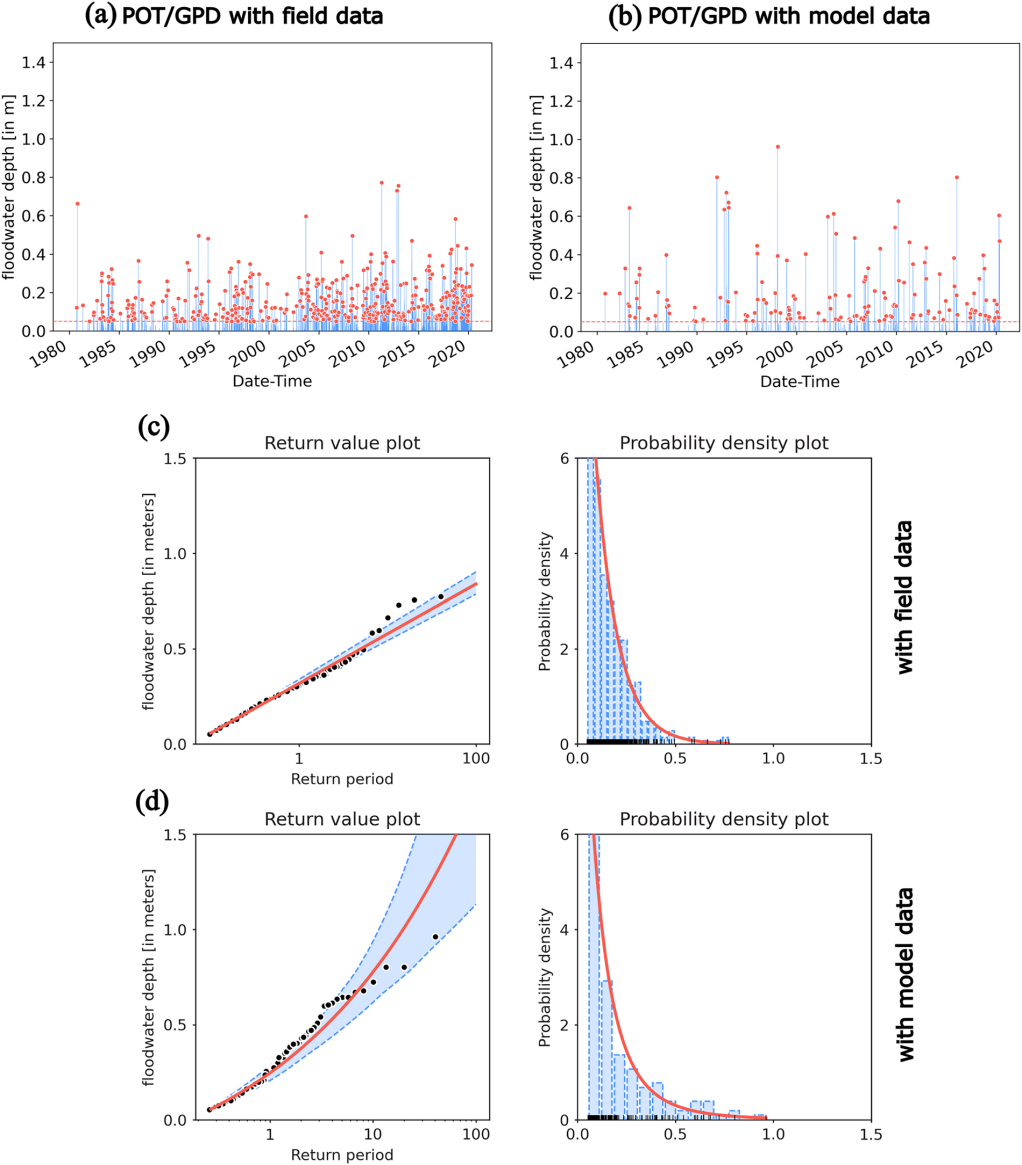
(**a**) Extreme events selected using a Floodwater Depth threshold of 5 cm (over peak tidal elevation) from 40-year period field dataset at Reedy Point, DE; (**b**) Extreme events selected using the model dataset; (**c**) return value estimate and probability distribution function (PDF) of the Floodwater Depth using POT/GPD based on field data; (**d**) return value estimate and PDF of the Floodwater Depth using POT/GPD based on model data.

In this section, we evaluate the goodness of fit of the parametric models against the empirical distribution derived from observed data. This assessment employs both qualitative and quantitative methods. Alongside graphical techniques such as Q-Q and P-P plots, we conducted two-sided Kolmogorov-Smirnov (KS) tests using field gauge data and datasets generated by the FVCOM model. These tests are essential for determining whether the GEV and GPD distributions adequately represent the Floodwater Depth return values. Additionally, we calculated the Root Mean Square Error (RMSE) between the empirical and theoretical cumulative distribution functions (CDFs). We structured our analysis into two segments. The first segment compares the performance of the BM/GEV and POT/GPD models in predicting CDFs at Reedy Point, DE (RP) based on field gauge and FVCOM data, as shown in Figs. 10 and 11. The error metrics from this comparison provide a quantitative assessment of model performance. The second segment presents a side-by-side comparison of return value estimates at the same location (Figs. 12 and 13).

For Floodwater Depth derived from field tide gauge data, the GEV model yielded a KS statistic of 0.07, an RMSE of 0.017, and a p-value of 0.97 (p > 0.05), indicating an excellent fit. Similarly, the GPD model produced a KS statistic of 0.02, an RMSE of 0.05, and a p-value of 0.94. When the same analysis was applied to Floodwater Depth estimates generated by the FVCOM model at the same gauge, the GEV model resulted in a KS statistic of 0.10, an RMSE of 0.04, and a p-value of 0.77, while the GPD model produced a KS statistic of 0.02, an RMSE of 0.05, and a p-value of 0.6. Overall, the results demonstrate that both the GEV and GPD distributions provide a robust fit to the observed data from both field measurements and FVCOM simulations. The CDF, Q-Q, and P-P plots (Figs. 10 and 11) further provide a qualitative assessment of goodness-of-fit between the empirical data and the fitted extreme value distributions. While both models align well with empirical distributions, larger deviations are observed in the tail region, particularly for quantiles based on the GPD and model-generated Floodwater Depth data. This visual assessment, coupled with the KS test results and RMSE values, confirms that the selected extreme value distributions effectively characterize the Floodwater Depth return levels.

After evaluating the parametric model performance, we estimated the Floodwater Depth return values for different periods (10, 25, 50, and 100) at the same site, RP. From Fig. 12a,b, we can see that there are more flood events in the field observations compared to model simulations; however, using the BM/GEV approach and selecting only the annual maxima (40 peaks), the pdf and the return levels estimated using both field and model data show comparable values (Fig. 12c,d). In Table 4, we also quantified the differences between return level estimates by BM/GEV. We observed that the model overestimates the 10, 25, 50, and 100-year return levels, where the error progressively increases from 0.09 m to 0.19 m. We want to point out that these estimates contradict the model’s tendency to underestimate total water levels during extreme events, as demonstrated in Table 2. In that table, we quantified error metrics such as bias, skill, and the correlation coefficient (R) based on hourly data collected over extended storm periods. These metrics reflect the model’s overall performance across the storm period, including flood and ebb phases, rather than focusing solely on the peak values. In Table 3, we further demonstrate that the model tends to underpredict tidal amplitudes, contributing to the overall negative bias during storm periods. However, when we isolate the maximum Floodwater Depth during storm periods – specifically, the peak values used in the return level analysis – the model overpredicts these peaks most of the time. Since the return level estimation relies on peak Floodwater Depth, we see the overestimation of extreme values in the return level estimates, even though the overall time series is biased low. The 95% confidence interval estimated from both datasets show a very similar range (shown in Fig. 12c,d); we provided the Floodwater Depth return level and 95% confidence interval limit for 10, 25, 50, and 100-year events in the dataset shared in the repository. For a similar comparison using the POT/GPD, in Fig. 13a,b, we clearly see that our long-term model simulation misses many smaller flood events at Reedy Point, DE, which lies in an estuary-river interaction zone. Even though the pdf shows a nearly similar distribution between field and model data (Fig. 13c,d), the lower sample size from the model simulation results in a larger 95% confidence interval as the return period increases. Overall, the difference in return level estimates using POT/GPD is greater compared to using BM/GEV (Table 4). For instance, for a 100-year event, the model data overpredicts the return level by 0.88 m with POT/GPD and by 0.19 m with BM/GEV.

**Table 4:** Comparison of BM/GEV and POT/GPD Floodwater Depth return level estimates using field and model data at Reedy Point, DE (RP).

Return Period (years)	floodwater depth (meters)			
BM/GEV	Field	Model	Difference	% Difference
10	0.58	0.67	0.09	15.5%
25	0.71	0.84	0.13	18.3%
50	0.81	0.97	0.16	19.7%
100	0.91	1.10	0.19	20.8%
**POT/GPD**				
10	0.58	0.78	0.20	34.4%
25	0.68	1.09	0.41	60.2%
50	0.76	1.38	0.62	81.5%
100	0.84	1.72	0.88	104.7%

The difference is estimated by subtracting field observation from the model.

**Fig. 14:**
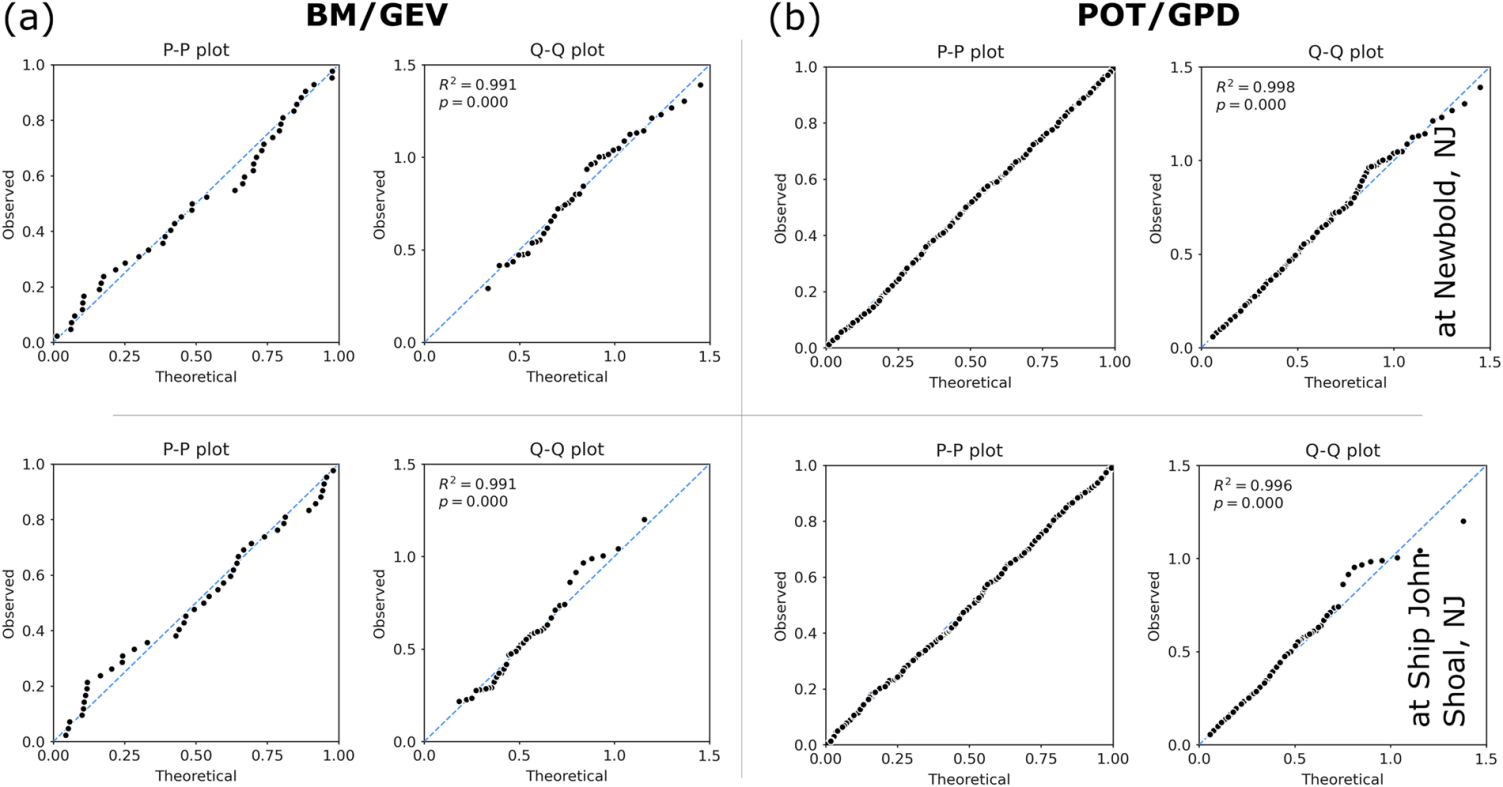
Comparison of the BM/GEV and POT/GPD model quality (based on FVCOM model data) using P-P and Q-Q plots in different parts of the estuary, Newbold, NJ, and Ship John Shoal, NJ.

**Fig. 15:**
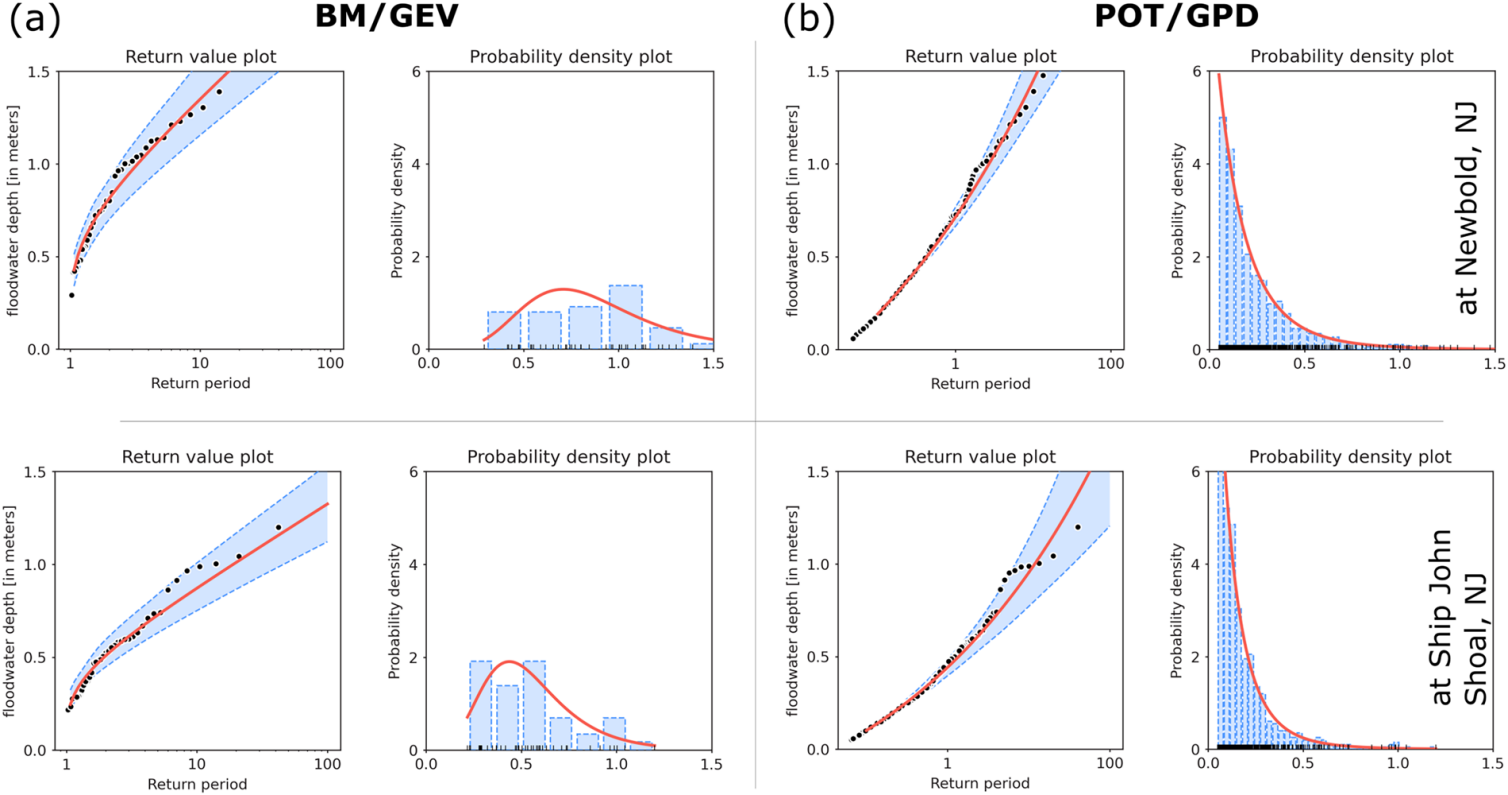
Comparison of return value estimate and probability distribution function of the Floodwater Depth using BM/GEV and POT/GPD (based on model data) in different parts of the estuary, Newbold, NJ, and Ship John Shoal, NJ.

Then, we compared the performance of the BM/GEV and POT/GPD models using FVCOM data at two other locations along the estuary, Newbold, NJ (NB) and Ship John Shoal, NJ (SJS), to evaluate how the statistical estimates, mainly the goodness of fit, return values and the probability distribution function can vary across different parts of the estuary (Figs. 14 and 15). Our study area is a complex region where the statistical estimates could vary based on the location and frequency of flood events, which are uniquely dominated by surge and fluvial discharge. The P-P and Q-Q plots in Fig. 14 supports our prior statement about the strength and weakness of these two EVA methods in estuarine extreme flood estimates. The theoretical distribution from BM/GEV, mainly the expected quantiles, shows a slightly better agreement with the empirical estimates compared to POT/GPD at SJS. Selecting model data progressively from the bay entrance to the upstream river increased the flood frequency for POT/GPD; however, it produced higher return values and wider confidence intervals compared to BM/GEV. At the same time, because of a small sample size of Floodwater Depth in the bay, both models have higher confidence intervals for large return periods (>25 years) while the range is smaller for the BM/GEV estimate. We provided a quantitative assessment of the return level difference between these two approaches at Newbold, NJ (NB) and Ship John Shoal, NJ (SJS) in Table 5. This comparison shows that although they both predict a nearly similar value for more frequent extremes, POT/GPD generates much larger return levels for 50- and 100-year events in both parts of the estuary, and users should be cautious about the uncertainties from both these statistical models.

**Table 5:** Comparison of BM/GEV and POT/GPD Floodwater Depth return level estimates using model data at two locations: Newbold, NJ (NB) and Ship John Shoal, NJ (SJS).

Return Period (years)	floodwater depth (meters)		
At Newbold, NJ (NB) (upstream river)	BM/GEV	POT/GPD	Difference
10	1.35	1.45	0.10
25	1.62	1.82	0.20
50	1.82	2.13	0.31
100	2.02	2.47	0.45
**At Ship John Shoal, NJ (SJS) (Bay)**			
10	0.87	0.95	0.08
25	1.05	1.22	0.17
50	1.19	1.45	0.26
100	1.33	1.72	0.39

## Usage Notes

The exceedance probability of Floodwater Depth and stillwater elevation (combining Floodwater Depth with peak tidal elevation), provided in the MSDlive repository^55^, can be valuable for various coastal decision-making applications. This data can help coastal planners and managers assess flood hazards and make informed decisions. In estuarine systems like DBR, where storm surge and river discharge can have varying impacts, this dataset is crucial for local management, especially in areas such as the bay region (characterized by low population density and rich natural systems like wetlands) and the urban environment (with higher population density in the City of Philadelphia). The multi-decadal time series of TWL and Floodwater Depth can assist stakeholders in understanding the dynamics and patterns of flooding. The estimate of stillwater elevation return levels can help create flood insurance maps and design coastal infrastructure, including housing, transportation, water and wastewater systems, electrical grids, storm protection structures, marsh restoration projects, and floodplain management. The dataset addresses a significant gap in the spatial availability of field data by providing high-resolution spatial information essential for assessing flood risk in urban areas like Philadelphia, where tidal influences and river flows converge. For example, the TWL time series from this dataset can be used as coastal forcing at the coastal-urban interface for urban flood modeling focused on the City of Philadelphia. The strength of the coupled high-resolution, process-based model used in this study is that it provides a more reliable prediction of coastal flooding - especially for the zones where complex flood dynamics are challenging to predict with large-scale coastal models or coastal models forced by sparse observations from gauges.

Furthermore, other researchers and engineers can utilize this dataset to model wave hazards, wetland and floodplain erosion, population dynamics, and risk assessment. It can also contribute to understanding long-term changes in estuarine dynamics, including the impact of sea-level rise on tidal processes throughout the entire system (bay and river). A comprehensive analysis involving sea-level rise, long-term climate forcings, and continuous fluvial and estuarine flooding could provide valuable insights into the influence of climate change on extreme flood events.

Despite its various usefulness, the dataset also has a few limitations that we need to acknowledge:

1. The model underpredicts peak TWL during extreme events by varying magnitudes at different parts of the estuary. Especially near the bay entrance and close to the shoreline (Lewes, DE), we observed an averaged model bias of −0.18 m. As we looked close to the river portion of the study area (e.g., Newbold, NJ), we observed a tidal constituent (M2) amplitude error of around −0.19 m, which reduced progressively near the bay entrance. These values suggest that when using the peak tidal elevation in the river discharge-dominated zone (RP to NB), we must consider the tidal amplitude error in the final stillwater elevation estimates. In the bay region (SJS to LW), it’s essential to carefully consider peak TWL errors when estimating Floodwater Depth return levels.
2. Our analysis used two classical extreme value analysis methods, BM/GEV and POT/GPD, to estimate the 10, 25, 50, and 100-year event Floodwater Depth and their confidence intervals. When comparing the return level estimates using field and model Floodwater Depth, we found that both statistical methods overpredicted the return level when using the model flood dataset. The overprediction was notably higher for POT/GPD. In this work, we did not delve into the underlying reasons behind the performance of both EVA methods, such as the sensitivity of sample size in BM/GEV or the threshold used in POT/GPD, leaving it as a topic for future study. Additionally, users can use a more refined and robust statistical methods, such as non-stationary extreme value models or Bayesian approaches, to estimate the return levels directly from the provided TWL or Floodwater Depth time series.
3. In our stillwater elevation return level estimates, we merged the peak tidal elevation in 2019 with Floodwater Depth levels. This peak tidal elevation included the purely tidal elevation and the adjusted mean sea level based on the historical sea-level trend until 2019. Many recent studies, based on projected SLR rates for different climate scenarios, provided the exceedance probability of extreme water levels that included future changes to the mean sea level. Including the future increase in the sea level can change the frequency and peak of the return levels, providing a different estimate of the extremes. However, the simplistic assumptions of future sea levels could work well near coastlines without the significant presence of tidal wetlands and floodplains. In converging tidal estuaries, like DBR, the along-channel storm tide (tide+surge) propagation could non-linearly interact with the estuarine morphology, and the Floodwater Depth could increase or decrease at various parts of the estuary depending on the tidal wetting and drying of the low-lying wetlands (further details are given in Deb *et al*.^27^). The analysis shows that, near PHL, while the mean sea level is expected to rise due to the increase in sea level, the Floodwater Depth for the same storm event could decrease in the future due to a higher rate of wetland submergence. The results from Deb *et al*.^27^ suggest that we should be careful about any linear superposition of future SLR conditions in estimating stillwater elevation return levels for this study area. Future work is needed to assess whether the salt marshes in the region will keep up with the projected increase in the mean sea level and how it changes the storm surge propagation along the tidal channel.
4. We provided the model results using 1681 point locations on the channel (water body) distributed over the bay and river regions. Model nodes or point locations over land go through wetting and drying, making them unsuitable for tidal harmonic analysis. Also, the reduced sample size of Floodwater Depth over land is not suitable for extreme value analysis.

## Data Availability

The 3D ocean model FVCOM code is available from the MEDM Lab (https://github.com/FVCOM-GitHub). The Distributed Hydrology Soil Vegetation Model (DHSVM) code is available at https://www.pnnl.gov/projects/distributed-hydrology-soil-vegetation-model. We performed tidal harmonic analysis using the T_Tide Harmonic Analysis Toolbox (https://www.eoas.ubc.ca/~rich/. Flood return levels are estimated using the Python-based stationary extreme value analysis tool called *PyExtreme* (github.com/georgebv/pyextremes). The details about Matlab’s ‘detrend’ function are provided in https://www.mathworks.com/help/ident/ref/iddata.detrend.html. The 2D color maps are produced using cmocean (https://github.com/matplotlib/cmocean).
